# Adaptive state-feedback echo state networks for temporal sequence learning

**DOI:** 10.1038/s41598-026-42971-5

**Published:** 2026-03-16

**Authors:** Codrin A. Lupascu, Daniel Coca

**Affiliations:** 1 Technical University “Gh. Asachi” Iasi, Iasi, Romania; 2 School of Engineering, Newcastle University, Newcastle upon Tyne, UK; 3 School of Electrical and Electronic Engineering, The University of Sheffield, Sheffield, UK

**Keywords:** Echo state networks, State-feedback learning, Extended Kalman filter, Reservoir computing, Dynamical systems modelling, Neural feedback control, Neuroscience, Computational neuroscience, Dynamical systems, Mathematics and computing, Computer science

## Abstract

Echo State Networks are recurrent neural networks that leverage a random reservoir’s dynamics, training only a simple readout layer. This approach is computationally efficient but limits network performance. By introducing state or output-feedback connections, the reservoir dynamics can be shaped to enhance the network’s ability to capture complex temporal dependencies. Here we propose AFRICO (Adaptive Feedback, Readout and Input with Connectivity Optimisation), a novel training framework for Echo State Networks that adapts input and state-feedback weights via an Extended Kalman Filter, followed by optimisation of a sparse readout layer that selectively connects reservoir states to the output. The key novelty lies in jointly adapting input and state-feedback pathways to shape reservoir dynamics, while separately constructing a sparse task-specific readout. This approach enables the Echo State Network to capture both the internal dynamics and output mapping of the target system. We evaluate AFRICO on synthetic linear and nonlinear systems, as well as in vivo electrophysiological recordings from fly photoreceptors, demonstrating its versatility for general-purpose input–output time-series modelling across both engineering and biological domains. Across varied hyperparameter initialisations, AFRICO achieves up to 88% reduction in Normalised Mean Squared Error compared to Echo State Networks with fixed output-feedback, while maintaining modest computational effort relative to fully trained Recurrent Neural Networks.

**Supplementary Information:**

The online version contains supplementary material available at 10.1038/s41598-026-42971-5.

## Introduction

Recurrent Neural Networks (RNNs)^1–4^ can learn and predict complex dynamical behaviours^5–7^, providing plausible models of brain architecture and computation^8^. Neural representations and dynamics often exhibit long temporal dependencies, which pose significant challenges for training conventional RNNs^9^. This has prompted development of novel network architectures and training algorithms designed to effectively learn and model long-range dependencies.

Echo State Networks (ESNs) address this challenge by using a randomly connected reservoir to transform the input data into a high-dimensional state-space representation. By training only the readout weights^10–13^, ESNs achieve comparable performance to fully trained RNNs at a fraction of the computational cost.

ESNs consist of a dynamic *reservoir*—an RNN with fixed, random weights - that maps the inputs $u:{\mathbb{R}} \to {\mathbb{R}}$ to a high-dimensional state-space ${\mathbf{x}}:{\mathbb{R}} \to {{\mathbb{R}}^n}$^14^, and a feedforward *readout*, with adjustable weights mapping the reservoir states ${\mathbf{x}}$ to the output $y:{\mathbb{R}} \to {\mathbb{R}}$. Since only the weights of the readout or output map are trained, an obvious advantage of Reservoir Computing (RC) is that training an ESN network is computationally efficient and fast compared to training all the parameters of the network^14^.

Extensions to the original ESN model include incorporating output or state-feedback, reservoirs with scale-free or small-world characteristics^15^, uniformly distributed poles with adaptive bias^16^, augmented complex ESNs featuring nonlinear readout layers^17^, and the echo state Gaussian process^18^. While reservoirs typically exhibit nonlinear dynamics paired with a linear readout, alternative architectures with linear dynamic reservoirs and nonlinear readouts have also been explored^19–22^.

In standard ESNs, readout weights are typically optimised via least-squares algorithms^12,13,23–25^. Optimising readout connectivity can significantly improve accuracy^26^, mimicking features of the brain’s architecture, where densely connected, static neurons handle general processing, and sparsely connected, plastic neurons support task-specific learning^27^. Black-box training approaches, such as full backpropagation through time (BPTT) in RNNs or LSTMs, offer greater flexibility but often lack interpretability and require extensive computational resources.

**Fig. 1:**
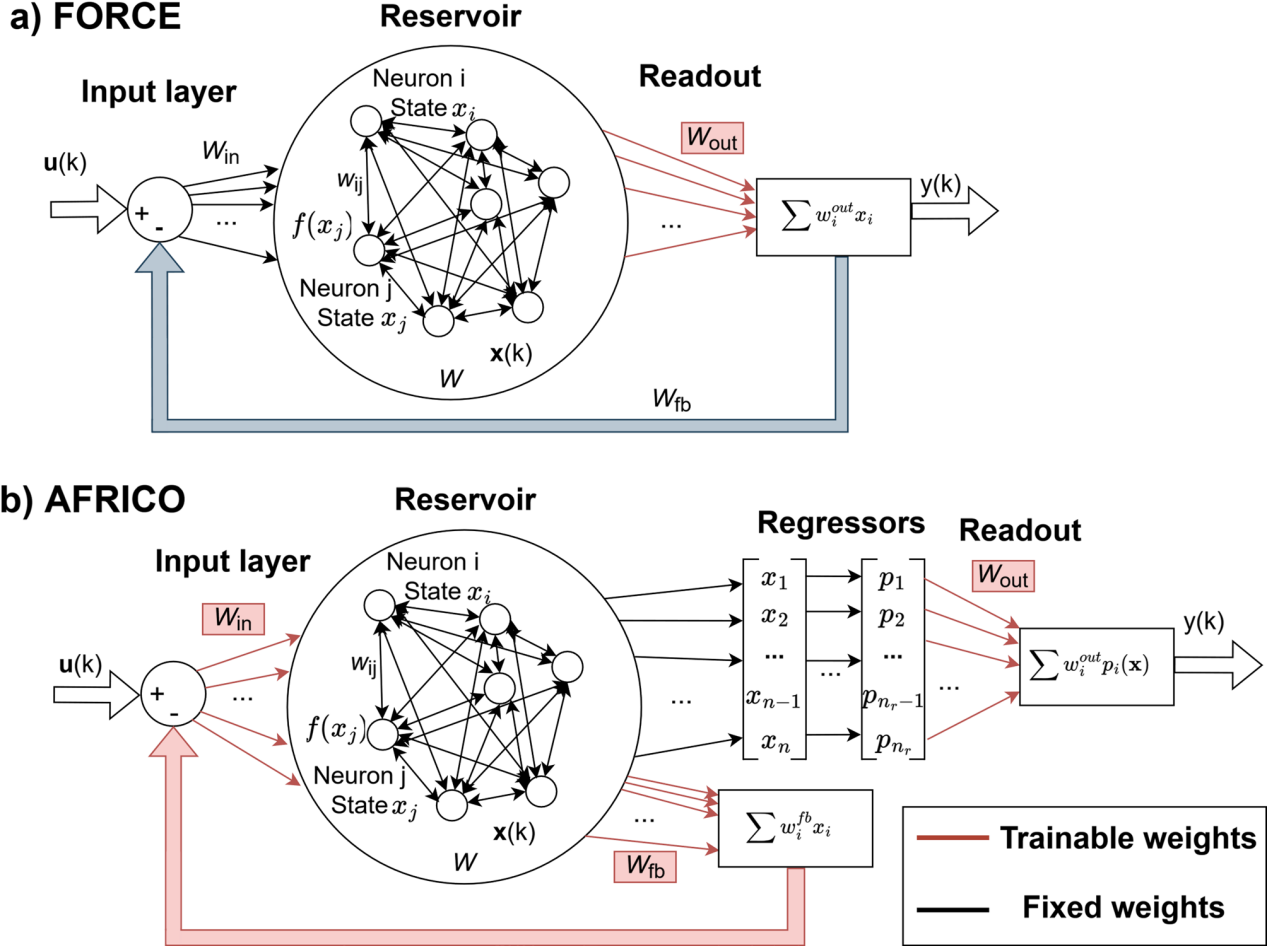
Architecture and adjustable weights for Echo State Networks with **a** fixed output-feedback FORCE and **b** novel state-feedback AFRICO. Trainable weights and pathways are shown in red.

Introducing an output-feedback loop with fixed weights (Fig. 1a) has been shown to improve ESN performance by shaping the reservoir dynamics while keeping only the readout weights trainable^12^. To mitigate instability caused by inaccurate early predictions, the target output is typically fed back during training. However, large initial errors can still destabilise learning^23^. The FORCE algorithm (First-Order Reduced and Controlled Error)^25^ addresses this by using recursive least squares (RLS) to continuously update the readout weights based on the true output.

Although fixed output-feedback improves temporal modelling by embedding task-specific dynamics into the reservoir^28,29^, optimising only the readout layer limits the network’s capacity to fully adapt to complex dynamics. In particular, holding the feedback weights fixed fails to exploit the potential for directly modulating the reservoir’s internal state evolution in a task-dependent manner.

Here we introduce AFRICO (Adaptive State-Feedback, Readout and Input with Connectivity Optimisation) a novel training method for ESNs with state-feedback, that jointly adapts the input and state-feedback weights using the Extended Kalman Filter (EKF), followed by a sparse readout optimisation using Orthogonal Forward Regression (OFR) with the Error Reduction (ERR) Criterion^30–32^. The proposed AFRICO architecture is illustrated in Fig. 1b alongside the conventional ESN with fixed output-feedback architecture used in FORCE where only the readout weights are trained, shown in Fig. 1a.

AFRICO addresses two core limitations in existing ESN frameworks: the lack of internal adaptation of reservoir dynamics and the rigid model structure and dense connectivity of a typical ESN readout which limits interpretability in tasks where knowing which internal states are ‘active’ may be valuable.

We provide a theoretical argument establishing that, under reservoir dimensionality matching between ESN and target system, adapting both input and feedback pathways is a necessary condition to replicate the input-output behaviour of a target dynamical system. This motivates the architectural choice and highlights the importance of dynamically shaping reservoir dynamics, especially where model size is constrained. ESNs with adaptive input, feedback, and readout weights retain the universal approximation property for causal, fading memory systems^13^. The inclusion of adaptive feedback further expands the network’s capacity to represent complex dynamics, consistent with results from nonlinear control theory.

AFRICO is evaluated across a number of synthetic system identification tasks, as well as in vivo electrophysiological recordings from fly photoreceptors^33^, which confirm that it consistently outperforms performance of ESN with fixed output feedback.

## Methods

In this section, we introduce the dynamical models considered in this study and define the notation used throughout the training and analysis procedures.

## Models

### Echo-state networks with output-feedback

A discrete-time ESN with *m* inputs, *N* units (neurons) with single output-feedback can be represented using the following state-space equations:

1 $$\begin{gathered} {\mathbf{x}}(k+1)=F\left( {W{\mathbf{x}}(k)+{W_{in}}{\mathbf{u}}(k) - {W_{fb}}y(k)} \right) \hfill \\ y(k)=H\left( {{\mathbf{x}}(k),{W_{out}}} \right) \hfill \\ \end{gathered}$$

Here ${\mathbf{u}}={\left[ {{u_1},...,{u_m}} \right]^T},{\mathbf{x}}={\left[ {{x_1},...,{x_N}} \right]^T},y$ denote the input, state and output vectors The function$$F={\left[ {\begin{array}{*{20}{c}} {f({x_1})}&{...}&{f({x_N})} \end{array}} \right]^T}$$, $f:{\mathbb{R}} \to {\mathbb{R}}$ represents the reservoir activation function, $H:{{\mathbb{R}}^N} \to {\mathbb{R}}$ is the output (readout) function, while $W \in {{\mathbb{R}}^{N \times N}}$, ${W_{in}} \in {{\mathbb{R}}^{N \times m}}$, ${W_{fb}} \in {{\mathbb{R}}^{N \times 1}}$ denote the reservoir, input-to-reservoir, output-feedback weight matrices respectively, and ${W_{out}} \in {{\mathbb{R}}^{1 \times N}}$is the output matrix. In this paper we consider linear and nonlinear (i.e. the hyperbolic tangent function) activation functions.

Generally, the readout function is expressed as $H({\mathbf{x}})={W_{out}}{\mathbf{x}}$^13^. However, the readout could also be a linear-in-the-parameters nonlinear map $H({\mathbf{x}})=\sum\nolimits_{i}^{{{n_r}}} {w_{i}^{{out}}{p_i}({\mathbf{x}})}$, where ${p_i}:{{\mathbb{R}}^N} \to {\mathbb{R}}$ are chosen as multivariate monomials, selected using a greedy algorithm from a complete polynomial model set of a specified order. This results in a readout function with sparse connectivity, compared to readouts implemented using radial basis functions^32^, for example.

### Echo-state networks with state-feedback

Consider the following ESN with state-feedback (Fig. 1b):

2 $$\begin{gathered} {\mathbf{x}}(k+1)=F\left( {(W+{W_{in}}{W_{fb}}){\mathbf{x}}(k)+{W_{in}}{\mathbf{u}}(k)} \right) \hfill \\ y(k)=H\left( {{\mathbf{x}}(k),{W_{out}}} \right)=\sum\limits_{{i=1}}^{{{n_r}}} {w_{i}^{{out}}{p_i}({\mathbf{x}}(k))} \hfill \\ \end{gathered}$$

As before, the same definitions as in (1) apply, ${W_{fb}} \in {{\mathbb{R}}^{l \times N}}$ is the state-feedback weight matrix. Equations (1) and (2) can be written in a more compact form as $y(k)=H\left( {F\left( {{\mathbf{x}}(k - 1),{\mathbf{u}}(k - 1),{W_{fb}},{W_{in}}} \right),{W_{out}}} \right)$.

For the general ESN model defined in (1) and (2), we prove the following lemma that establishes a necessary condition for input–output matching of a target system under dimensionality constraints, and it motivates the joint adaptation of input and feedback weights in the AFRICO framework.

#### **Lemma 1**

*For an ESN with state- or output-feedback*,* it is necessary to train the input and feedback weights*,* alongside the readout*,* in order to reproduce the local input–output behaviour of a target dynamical system*,* when both systems have the same state and output dimensions.*

#### Proof

See Supplementary Material Note 1.

Lemma 1 establishes a necessary condition under which an ESN with output- or state-feedback can locally replicate the input–output behaviour of a target system, assuming both systems are of equal dimension and admit linear approximations around a common, stable equilibrium point. It highlights that under matching reservoir conditions, fixed-feedback ESNs cannot match the target unless adaptive mechanisms are introduced, and motivates the proposed training framework.

Although the lemma does not provide universal approximation guarantees under the constraints imposed on the reservoir dimension, as demonstrated in^28^, an input-affine nonlinear ESN

3 $$\begin{gathered} {\mathbf{x}}(k)=F\left( {{\mathbf{x}}(k - 1)+M({\mathbf{x}}(k - 1)){\mathbf{u}}(k - 1)} \right) \hfill \\ y(k)=H\left( {{\mathbf{x}}(k)} \right) \hfill \\ \end{gathered}$$

can replicate the dynamics of an arbitrary nonlinear system described by the input-output equation

4 $$y(k)=G\left( {y(k - 1),y(k - 2),...,y(k - n)} \right)+{\mathbf{u}}(k)$$

by finding suitable state-feedback ${\mathbf{u}}'=K\left( {{\mathbf{x}}(k),{\mathbf{u}}(k)} \right)$ and readout $H({\mathbf{x}}(k))$ functions. If the target system is well-approximated by a local linear model, training the linear state-feedback gains can shape the reservoir dynamics to match the input-output behaviour. This would be reflected in the convergence of the feedback and input gains during online adaptation.

The dimensionality matching condition is not meant to reflect typical ESN configurations. It highlights that the ESN with adaptive input and feedback gains enable implementing the minimal architecture capable of replicating the dynamics of a system with matching state dimension. In practice, however, many systems exhibit low-dimensional attractors or admit reduced-order models, allowing accurate input–output approximation using a lower-dimensional reservoir that captures only the dominant modes of the target dynamics.

Specifically, the objective is to learn the weights ${W_{out}},{W_{in}},{W_{fb}}$ that minimise the empirical risk:

5 $$\mathop {\arg \hbox{min} }\limits_{{{W_{in}},{W_{fb}},{W_{out}}}} \frac{1}{N}\sum\limits_{{k=1}}^{N} {{{\left( {z(k) - H(F({\mathbf{x}}(k - 1),{\mathbf{u}}(k - 1),{W_{fb}},{W_{in}}),{W_{out}})} \right)}^2}}$$

based on input–output observations $\{ {\mathbf{u}}(1),...,{\mathbf{u}}(N)\} ,\{ z(1),...,z(N)\}$ generated by a target dynamical system:

6 $$\begin{gathered} {\mathbf{\bar {x}}}(k)=\bar {F}({\mathbf{\bar {x}}}(k - 1),{\mathbf{u}}(k - 1)) \hfill \\ z(k)=\bar {H}({\mathbf{\bar {x}}}(k)) \hfill \\ \end{gathered}$$

In contrast to standard methods such as FORCE, which adapt only the readout weights via output feedback, AFRICO optimises the state feedback and input weights, and then the readout in a two-stage procedure. The input weights determine how external signals project into the reservoir state space, shaping the input–state transfer characteristics and influencing the controllability of the system. In contrast, the feedback gains act analogously to state-feedback control in dynamical systems: they introduce a closed-loop shaping of the reservoir dynamics by altering the effective state-transition map without modifying the reservoir’s weights. This allows AFRICO to adapt both how external signals enter the system and how internal dynamics are regulated, thereby improving the model’s capability to approximate a broad class of target systems, even under limited reservoir capacity. Whereas FORCE adjusts only the readout weights while keeping both input and feedback fixed, a strategy that can introduce instability when the predicted output deviates significantly from the ground truth, particularly in early training or under noisy conditions, AFRICO employs a state-feedback formulation with recursive adaptation through the EKF. Additionally, AFRICO incorporates a sparse and interpretable readout using OFR, which further enhances generalisation while reducing model complexity. These distinctions are particularly relevant in low-resource settings.

It is important to highlight that the ESN architecture underpinning AFRICO satisfies the foundational universality condition in^13^ but also that incorporating adaptive input and feedback pathways, further expands the representational power of ESNs, as highlighted in^28^.

## Training

The proposed AFRICO algorithm optimises the input layer and state-feedback weights using the EKF algorithm^34^, a recursive estimation technique particularly well suited for systems with fixed internal dynamics and a limited set of trainable parameters, such as ESNs.

Unlike Stochastic Gradient Descent (SGD) or BPTT, which require unfolding the network over time and computing gradients through deep recurrent dependencies, EKF performs sequential updates based on local linearisation of the system dynamics. It estimates parameters by propagating uncertainty via a covariance matrix, incorporating both process and measurement noise into the update step. This structure allows the adaptation to remain responsive to the evolving system state while maintaining robustness in the presence of noise and temporal dependencies. EKF also avoids many of the stability and tuning challenges associated with SGD in recurrent settings, such as gradient vanishing, exploding, or sensitivity to the learning rate.

To incorporate trainable parameters, the EKF is applied to an augmented state vector that includes the reservoir state alongside the input and state-feedback weights. This formulation enables the algorithm to estimate the system’s latent state while simultaneously adapting the input pathways and feedback dynamics. The EKF is used solely during training to estimate the input and feedback weights, leaving the fixed recurrent reservoir unchanged. Rather than modifying internal connections directly, it adaptively shapes the effective state-transition behaviour of the network through the external input and feedback mappings. This allows the ESN to more closely reproduce the temporal structure of the target system.

In the second training stage, AFRICO employs a greedy OFR strategy^26^, to construct a sparse polynomial readout. This method incrementally selects monomials from a predefined candidate polynomial model set - including terms up to a specified maximum order - that most reduce the empirical risk defined in Eq. (5). Importantly, each selected monomial is a function of one or more specific state variables, meaning the term selection process also serves as a data-driven mechanism for identifying effective reservoir-to-output connectivity. This results in a sparse, task-specific readout structure that selectively decodes the most informative interactions among reservoir states. The maximum order can be increased iteratively during model construction, allowing the readout to adaptively explore higher-order interactions only when supported by the data. To mitigate overfitting, the selection process is guided by validation-based stopping criteria that limit model complexity based on predictive performance.

Compared to feature selection approaches such as LASSO, which optimises over all coefficients with L1 regularisation, and exhaustive subset selection methods, greedy forward regression offers a favourable trade-off between computational efficiency and approximation accuracy.

Stability in AFRICO-trained Echo State Networks is achieved through two complementary mechanisms. First, the reservoir itself is initialised to satisfy the Echo State Property by constraining the spectral radius of the recurrent weight matrix below unity (e.g., ρ(W) < 0.9), ensuring asymptotically stable open-loop dynamics. Second, the EKF-based adaptation of input and state-feedback weights incorporates process and measurement noise through its covariance updates. This serves as an implicit regulariser, constraining parameter updates in response to uncertain or noisy observations and promoting stability during training. In the current implementation, no explicit weight regularisation (e.g., L2 penalties) was applied during EKF updates and we observed no evidence of overfitting across multiple tasks. The framework remains compatible with regularisation techniques, which could be incorporated in future extensions.

While the current framework does not offer formal stability guarantees, empirical results suggest that the EKF-based adaptation of input and state-feedback weights can yield stable closed-loop dynamics under a broad range of conditions. In principle, if the target system is asymptotically stable and observable from its output, and noise covariances are well specified, the EKF-based adaptation converges to a parameter configuration that preserves stability^35^.

In this implementation of AFRICO, the EKF covariance matrix is initialised as a scaled identity matrix, *αI* where the scaling parameter *α* reflects an initial uncertainty over the trainable parameters. This choice provides a tunable prior that balances sensitivity to early data with numerical stability. Empirically, we found that moderate values of the scaling parameter (e.g., *α* = 1) support stable convergence across a wide range of tasks without requiring problem-specific tuning.

**Fig. 2:**
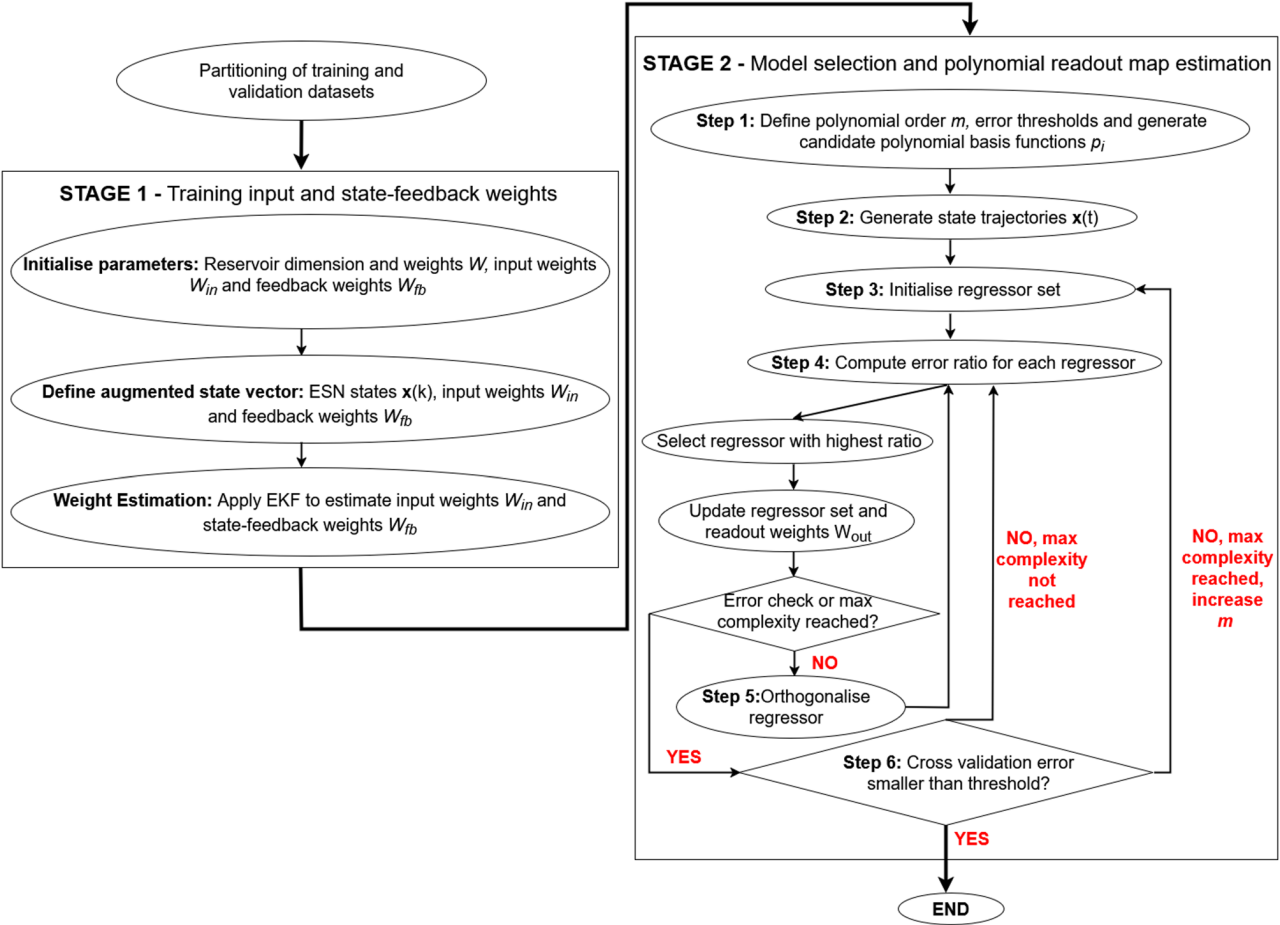
Flowchart of the AFRICO training procedure.

A flowchart illustrating the AFRICO algorithm is provided in Fig. 2, summarising the key steps described in the mathematical formulation in Supplementary Material Note 2.

## Results

The performance of AFRICO-trained ESNs with state-feedback was evaluated and compared to FORCE-trained ESNs with output-feedback^10^, using synthetic data generated from linear and nonlinear dynamical systems, as well as in vivo electrophysiological recordings of fly photoreceptor responses to naturalistic light stimuli^36^. Numerical studies explored ESNs with linear and nonlinear fixed reservoirs, analysing the impact of reservoir dimensionality and measurement noise on the accuracy of the AFRICO and FORCE frameworks. In all experiments, ESNs with fully connected reservoirs were randomly initialised for each simulation. The input, reservoir, and state-feedback weight matrices were initialised as dense and asymmetric, with entries drawn uniformly from the range [− 1, 1] The nonlinear reservoir neurons use the hyperbolic tangent activation function. The initial reservoir state was set to zero for all experiments, following standard practice in reservoir computing^24^. To evaluate robustness under noise, three measurement noise levels were considered, corresponding to Signal-to-Noise Ratios (SNRs) of 10 (high noise), 25 (medium noise), and 50 (low noise). Normalised Mean Squared Error (NMSE) is used to evaluate the performance of the models as it provides a scale-invariant measure of prediction accuracy. This allows for a fair comparison across models and datasets with differing signal magnitudes.

### Example A: Linear dynamical system

Consider the stochastic linear discrete-time system:

7 $$\begin{gathered} {\mathbf{x}}(k+1)={A_T}{\mathbf{x}}(k)+{B_T}{\mathbf{u}}(k) \hfill \\ y(k)={C_T}{\mathbf{x}}(k)+\chi (k) \hfill \\ \end{gathered}$$

where ${A_T},{B_T},{C_T}$ are (*N*,* N)*, (*N*,1) and (1,*N*) matrices respectively, **x** is the (*N*,1) state vector; *u* and *y* are the input and output respectively and *χ* is a normally distributed white noise with zero mean and variance *σ*^2^ = 1. The input is drawn from a uniform distribution in the interval [0,1].

The system described in (7) was simulated using an i.i.d. input sequence **u**(*k*), uniformly distributed in (0,1). The system matrices were randomly generated for each simulation instance, with the spectral radius of the system matrix **A** satisfying *ρ*(**A**) < 0.2.

The ESNs with output-feedback (Fig. 1a) were trained using the FORCE algorithm, while the ESNs with state-feedback (Fig. 1b) were trained using the AFRICO algorithm. The reservoirs were randomly initialised across 100 independent instances, each with a uniformly distributed eigenspectrum and a spectral radius satisfying 0.8 < *ρ*(*W*) < 0.9. Several dimensionalities have been tested, ranging between 20 and 80 neurons. The readout layer was selected to be linear. The training data consists of 1800 points, while the validation data consists of 400 points.

Although it is not standard practice to initialise a reservoir with a ‘narrow’ eigenspectrum, we adopt this setting to highlight that when the eigenvalues are distributed within a bounded spectral radius that constrains the reservoir’s dynamic range, learning the correct input-output behaviour becomes significantly more challenging. This highlights the advantage of state-feedback learning, as it allows the reservoir to adapt its effective dynamics, compensating for the limitations imposed by the initial eigenvalue distribution. By training ESNs in this constrained setting, we demonstrate that adaptive state-feedback mechanisms can recover functional behaviour even when the reservoir’s intrinsic eigenspectrum is suboptimal.

**Fig. 3:**
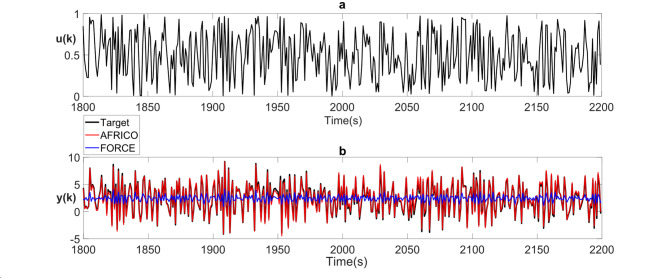
Example A: **a** input and **b** model predicted output for AFRICO-trained ESNs with state-feedback and linear reservoirs (red), FORCE-trained ESNs with output-feedback and nonlinear reservoirs (blue), superimposed on the target output (black), *N* = 80 neurons, SNR of 25, averaged over 100 simulations.

Figure 3 illustrates the closed-loop response of the AFRICO model compared to an ESN without state-feedback. The AFRICO-trained network closely tracks the target trajectory, while the FORCE-trained variant exhibits significant deviation. This highlights the critical role of adaptive state-feedback in shaping the reservoir’s internal dynamics for accurate long-term prediction.

The performance gap observed in Fig. 3 reflects a structural limitation of output-feedback ESNs, rather than from insufficient FORCE training or instability. When the reservoir eigenspectrum is narrowly distributed, the internal dynamics are inherently constrained. FORCE employs fixed output-feedback gains that project only through the output space, which has rank limited to the output dimension. This rank limitation constrains closed-loop shaping of the reservoir dynamics and, in general, precludes arbitrary pole placement with static output feedback. In contrast, AFRICO adapts a full state-feedback gain matrix via the EKF, providing high-dimensional internal modulation of reservoir trajectories. This mechanism enables AFRICO to recover accurate input–output behaviour even under suboptimal reservoir initialisation, consistent nonlinear control theory insights and universal approximation results for fading memory systems^13,28^.

In terms of approximation accuracy, ESNs trained using the AFRICO algorithm consistently outperform traditional output-feedback ESNs trained with FORCE. For instance, with a reservoir size of *N* = 80 and a SNR of 25, the AFRICO-trained model achieved a significantly lower NMSE of 0.05, compared to 0.43 for the FORCE-trained counterpart. Comparable improvements were also observed across other configurations, including *N* = 20 and *N* = 40, under both moderate and high noise conditions (SNR = 25; 10).

**Fig. 4:**
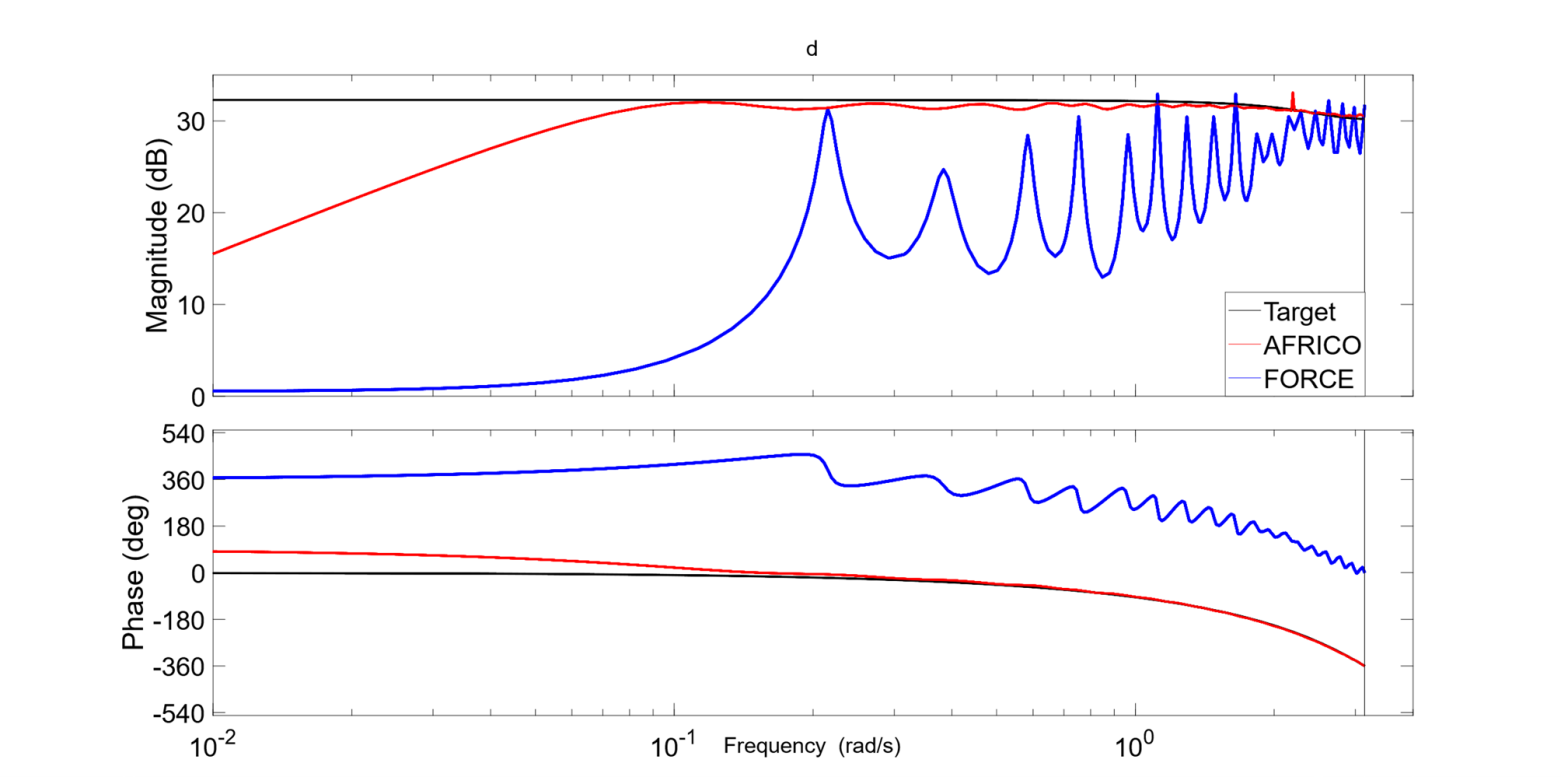
Example A: Magnitude and phase plots of the frequency response function of the target system (black), compared with the averaged magnitude and phase of the frequency responses of the AFRICO-trained ESN models (red) and the FORCE-trained ESN models (blue) with *N* = 80 neurons, SNR of 25, based on 100 simulations.

A comparison of the frequency response function of the target linear system (7) with the averaged responses of ESNs trained using AFRICO and FORCE (Fig. 4) reveals that the AFRICO-trained models more accurately reproduce the target system’s magnitude and phase characteristics compared to models trained using fixed output-feedback under the FORCE algorithm.

**Fig. 5:**
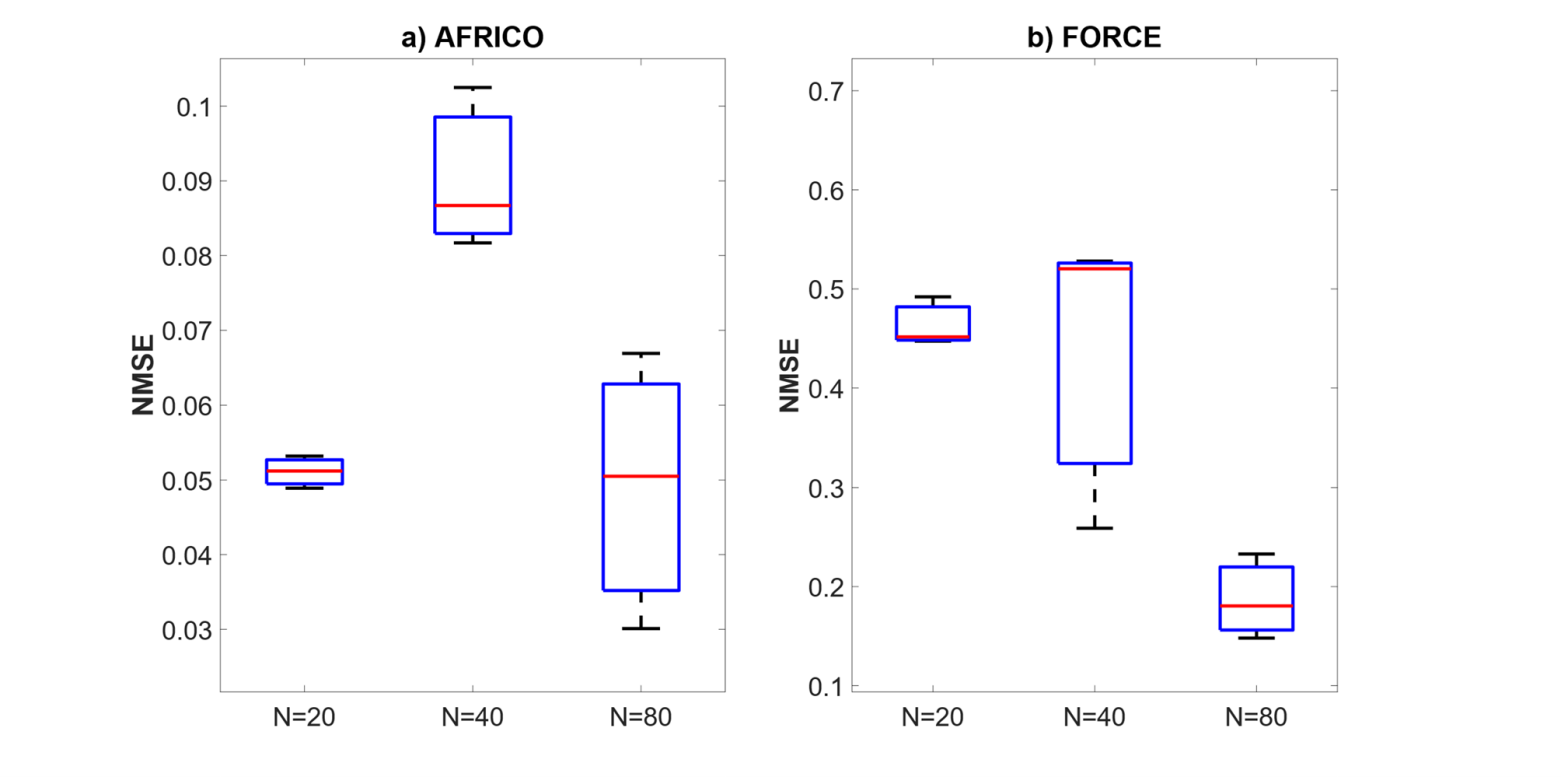
Example A: Boxplots showing the distribution of standard prediction errors for AFRICO and FORCE-trained ESNs across varying reservoir sizes, SNR of 25. Each box represents 100 independent simulations per configuration using **a** AFRICO-trained ESNs with state-feedback and **b** FORCE-trained ESNs with output-feedback.

**Fig. 6:**
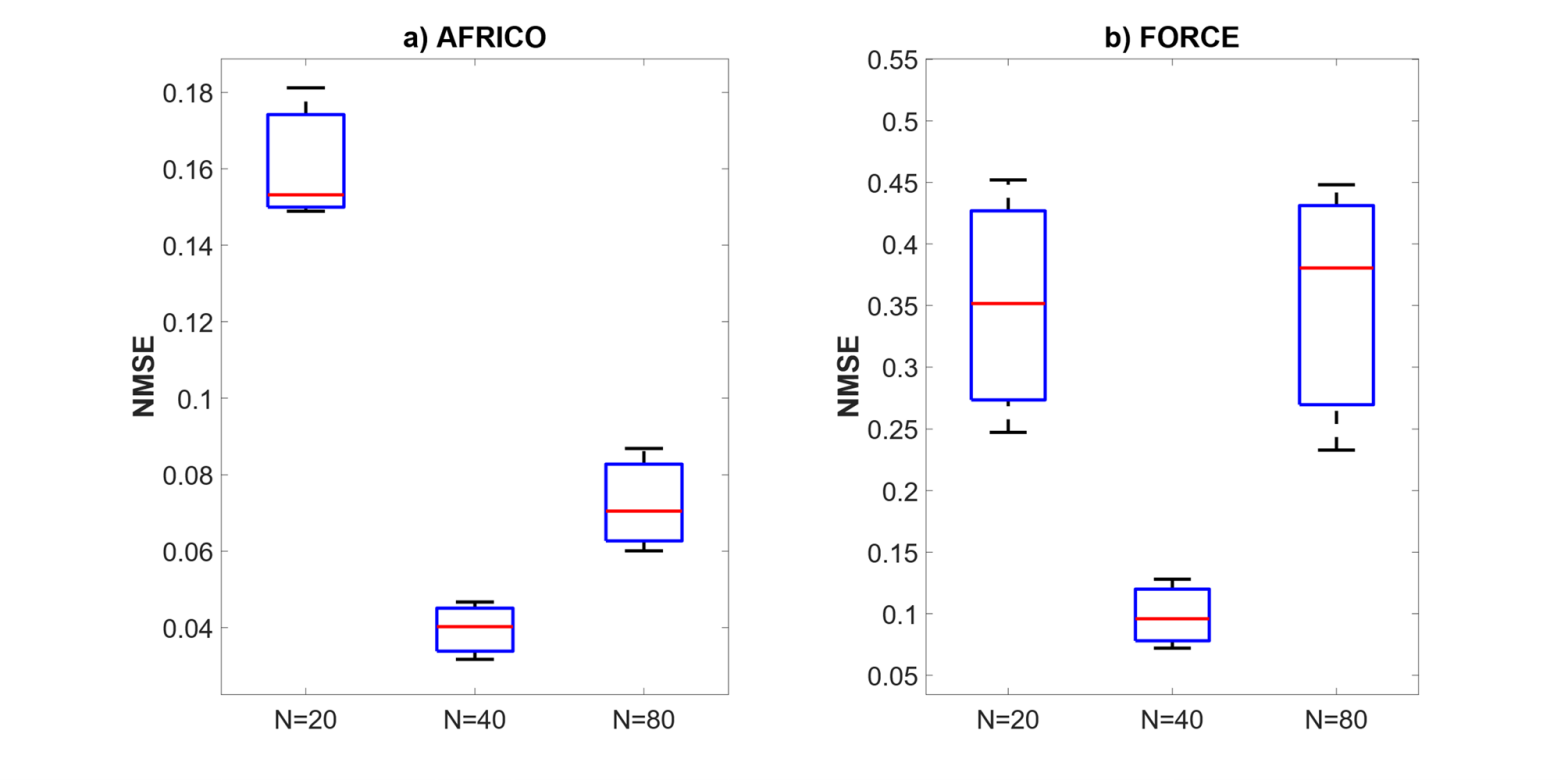
Example A: Boxplots showing the distribution of standard prediction errors for AFRICO and FORCE-trained ESNs across varying reservoir sizes, SNR of 10. Each box represents 100 independent simulations per configuration using **a** AFRICO-trained ESNs with state-feedback and **b** FORCE-trained ESNs with output-feedback.

**Fig. 7:**
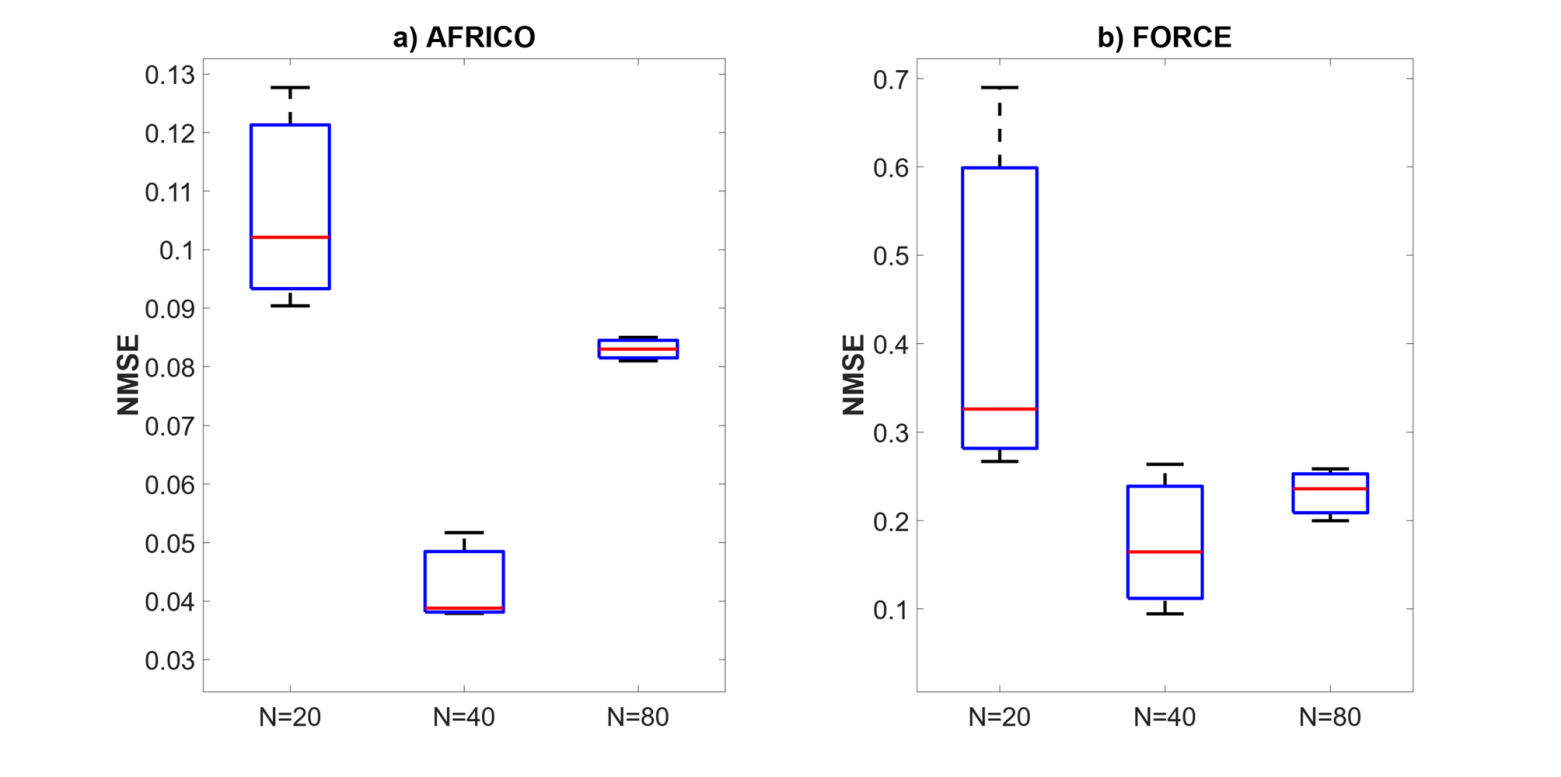
Example A: Boxplots showing the distribution of standard prediction errors for AFRICO and FORCE-trained ESNs across varying reservoir sizes, SNR of 50. Each box represents 100 independent simulations per configuration using **a** AFRICO-trained ESNs with state-feedback and **b** FORCE-trained ESNs with output-feedback.

Figure 5 compares the NMSE distributions of AFRICO and FORCE across varying reservoir sizes. AFRICO consistently achieves lower median NMSE than FORCE at all sizes, with notably tight error distributions at *N* = 20, indicating strong robustness to initialisation and compact reservoir configurations. At *N* = 80, AFRICO reaches its best median performance, though the variance increases, suggesting some sensitivity to initialisation. In contrast, FORCE exhibits consistently higher NMSE and greater variability, particularly at *N* = 40, where performance is highly unstable across runs. While FORCE shows improved accuracy at *N* = 80, its median error remains significantly higher than AFRICO’s, underscoring the advantage of adaptive feedback in enabling both accurate and efficient modelling. Similar trends were observed for other SNRs (high noise − 10 and low noise − 50), as illustrated in Figs. 6 and 7.

### Example B: Synthetic linear-nonlinear target system

Consider the following stochastic nonlinear discrete-time target system:

8 $$\begin{gathered} {\mathbf{x}}(k+1)={A_T}{\mathbf{x}}(k)+{B_T}{\mathbf{u}}(k) \hfill \\ y(k)=\sum\limits_{{i=1}}^{{39}} {[0.5{{({x_{i+1}} - x_{i}^{2})}^2}+{{(0.5 - {x_i})}^2}]} +\chi (k) \hfill \\ \end{gathered}$$

with the same definitions as in (7).

**Fig. 8:**
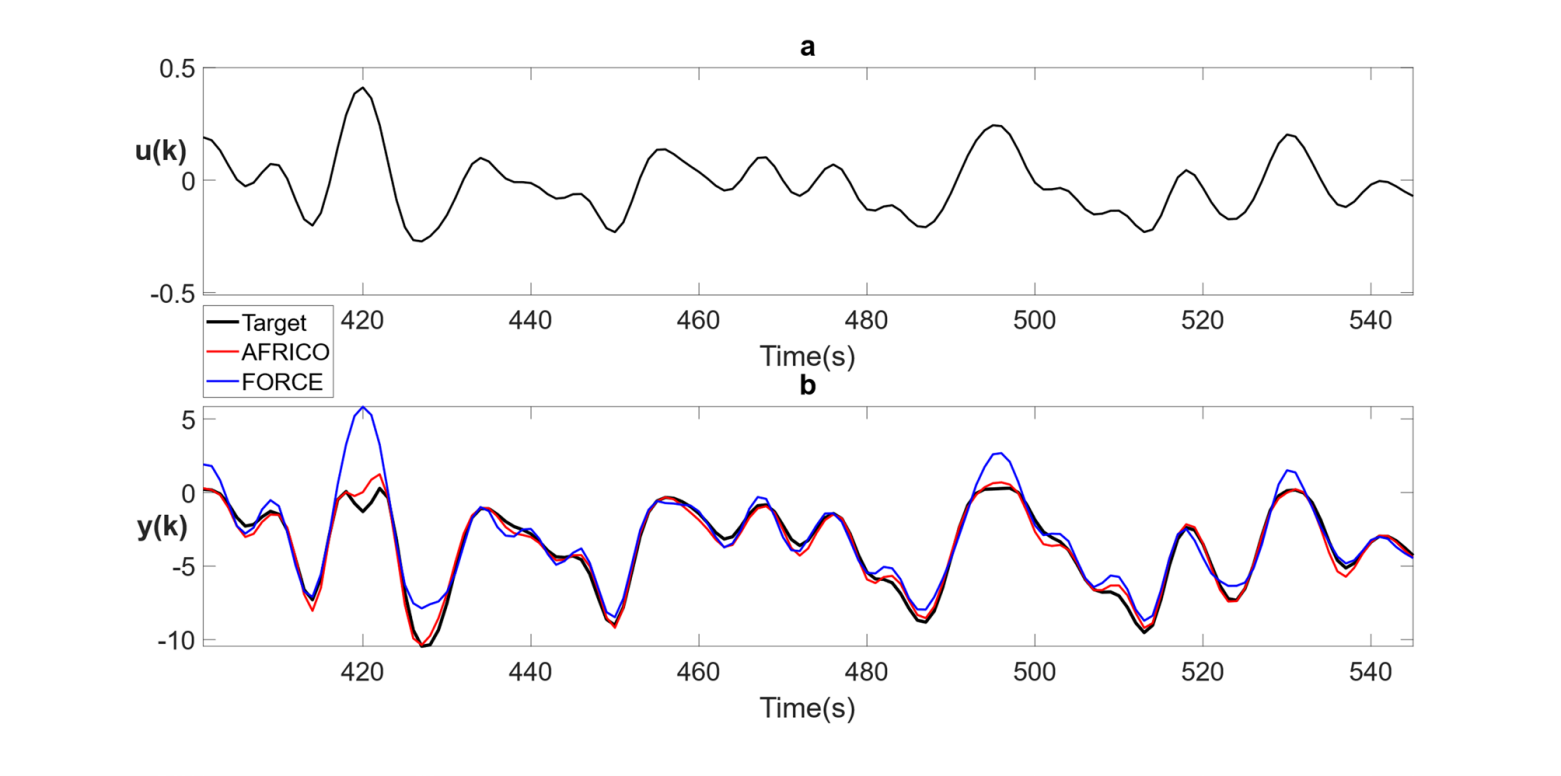
Example B: **a** input and **b** model predicted output for AFRICO-trained ESNs with state-feedback and linear reservoirs (red), FORCE-trained ESNs with output-feedback and nonlinear reservoirs (blue), superimposed on the target output (black), *N* = 80 neurons, SNR of 25, averaged over 100 simulations.

Standard initialisation of the hyperparameters across 100 independent instances, fully-connected reservoirs with a uniformly distributed eigenspectrum and a spectral radius *ρ*(*W*) < 0.9, guaranteeing open-loop stability, have been considered. Several dimensionalities have been tested, ranging between 20 and 80 neurons. The readout function was modelled as a polynomial function, with terms selected using Orthogonal Forward Regression with the Error Reduction criterion, from a candidate model set of polynomial regressors, up to cubic order (91,880 candidate terms for *N* = 80). The training data consists of 400 points, whilst the validation/testing data consists of 150 points (Fig. 8).

An illustrative set of polynomial readout terms selected across several runs for a linear reservoir with *N* = 80 is $\{ {x_2},{x_4},{x_1}{x_3},x_{4}^{2},{x_2}{x_5},x_{3}^{2},{x_1}{x_4},x_{2}^{2}{x_3},x_{1}^{3}\}$ corresponding to ~ 1–2% of the candidate model set of 91,880 consisting of all monomials up to cubic order. The exact terms vary with random initialisation, but the overall sparse structure is consistent. Here ${x_i}$ denotes the *i*th reservoir state.

**Fig. 9:**
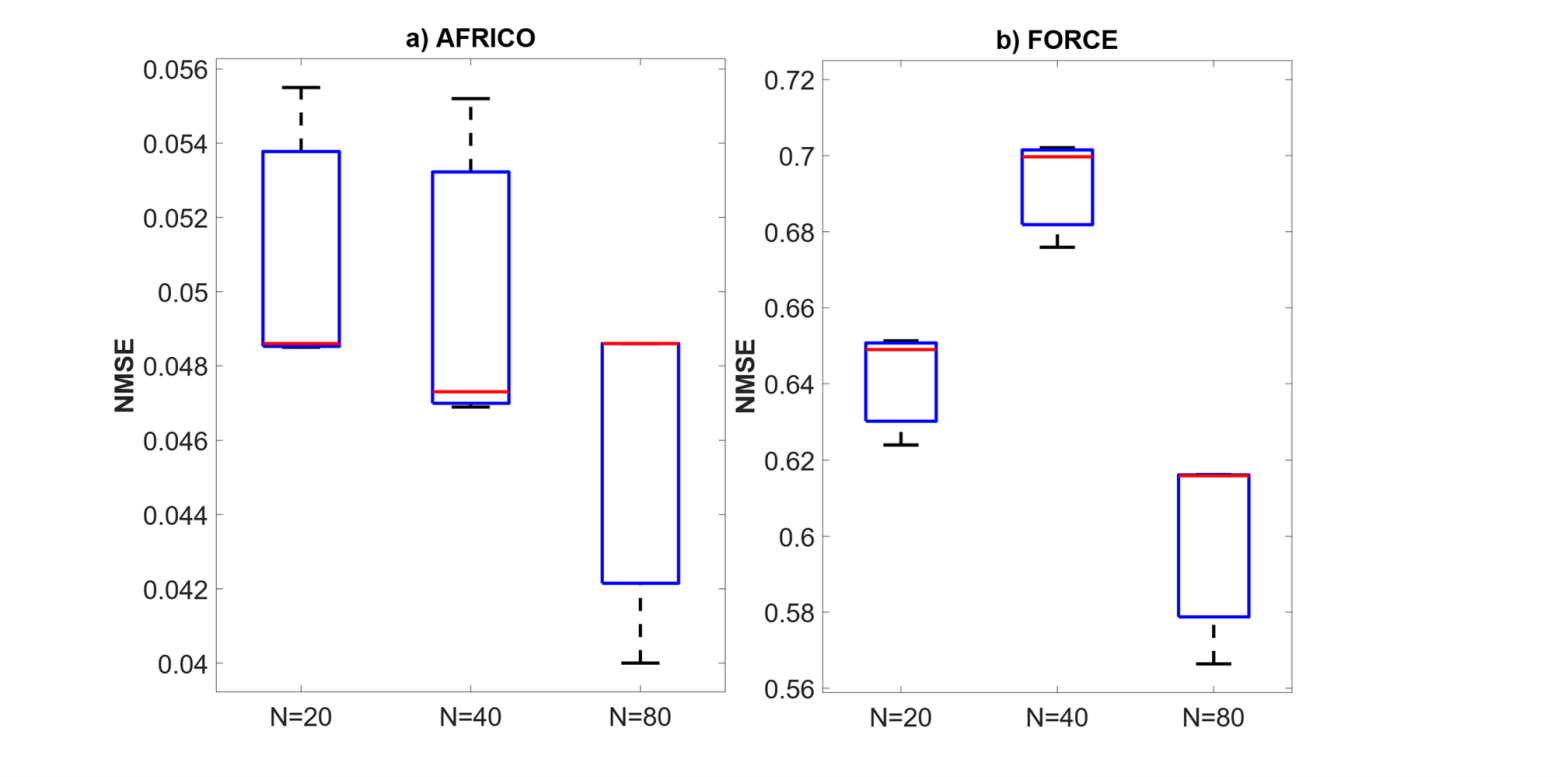
Example B: Boxplots showing the distribution of standard prediction errors for AFRICO and FORCE-trained ESNs across varying reservoir sizes, SNR of 25. Each box represents 100 independent simulations per configuration using **a** AFRICO-trained ESNs with state-feedback and **b** FORCE-trained ESNs with output-feedback.

**Fig. 10:**
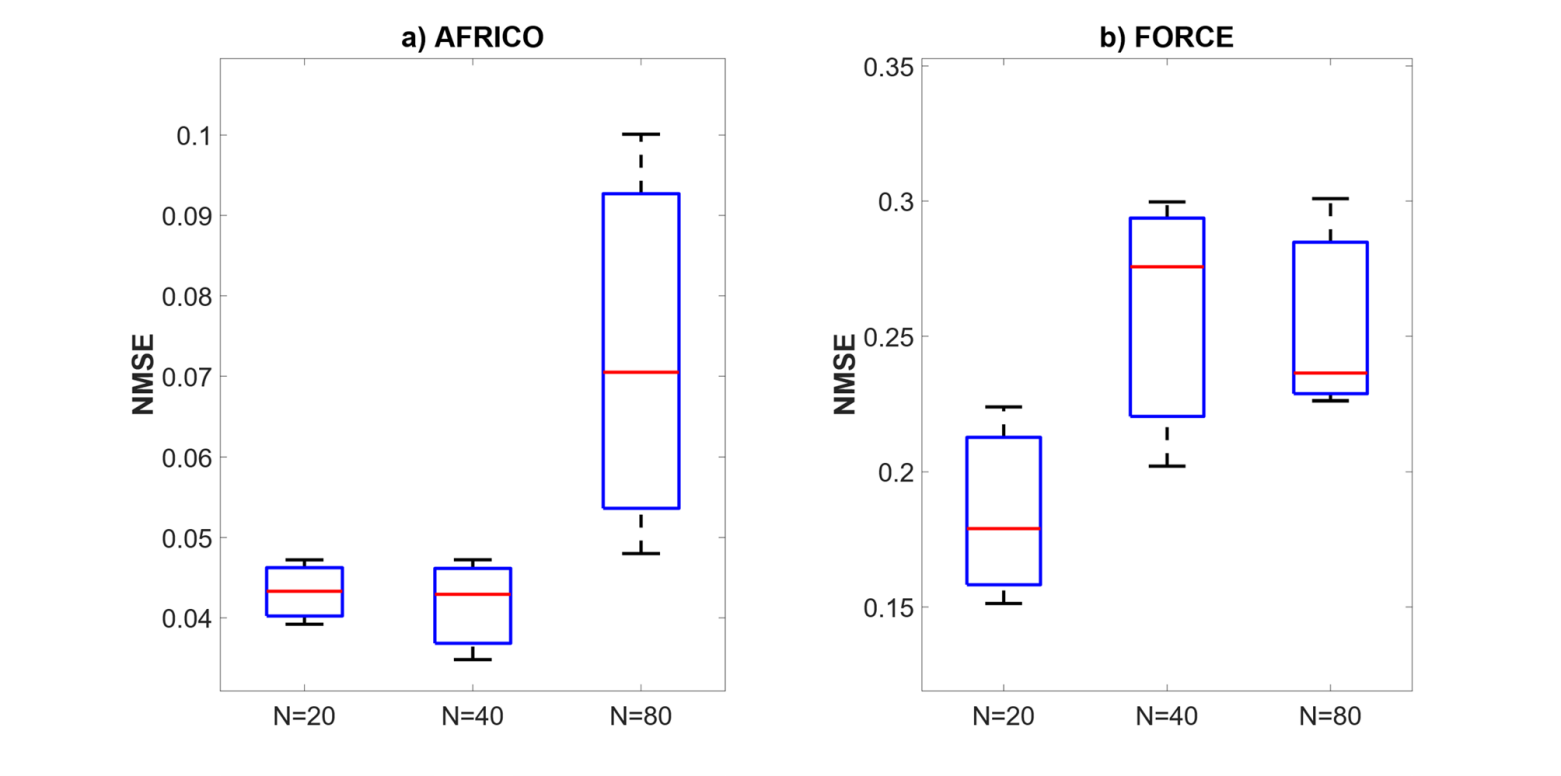
Example B: Boxplots showing the distribution of standard prediction errors for AFRICO and FORCE-trained ESNs across varying reservoir sizes, SNR of 10. Each box represents 100 independent simulations per configuration using **a** AFRICO-trained ESNs with state-feedback and **b** FORCE-trained ESNs with output-feedback.

**Fig. 11:**
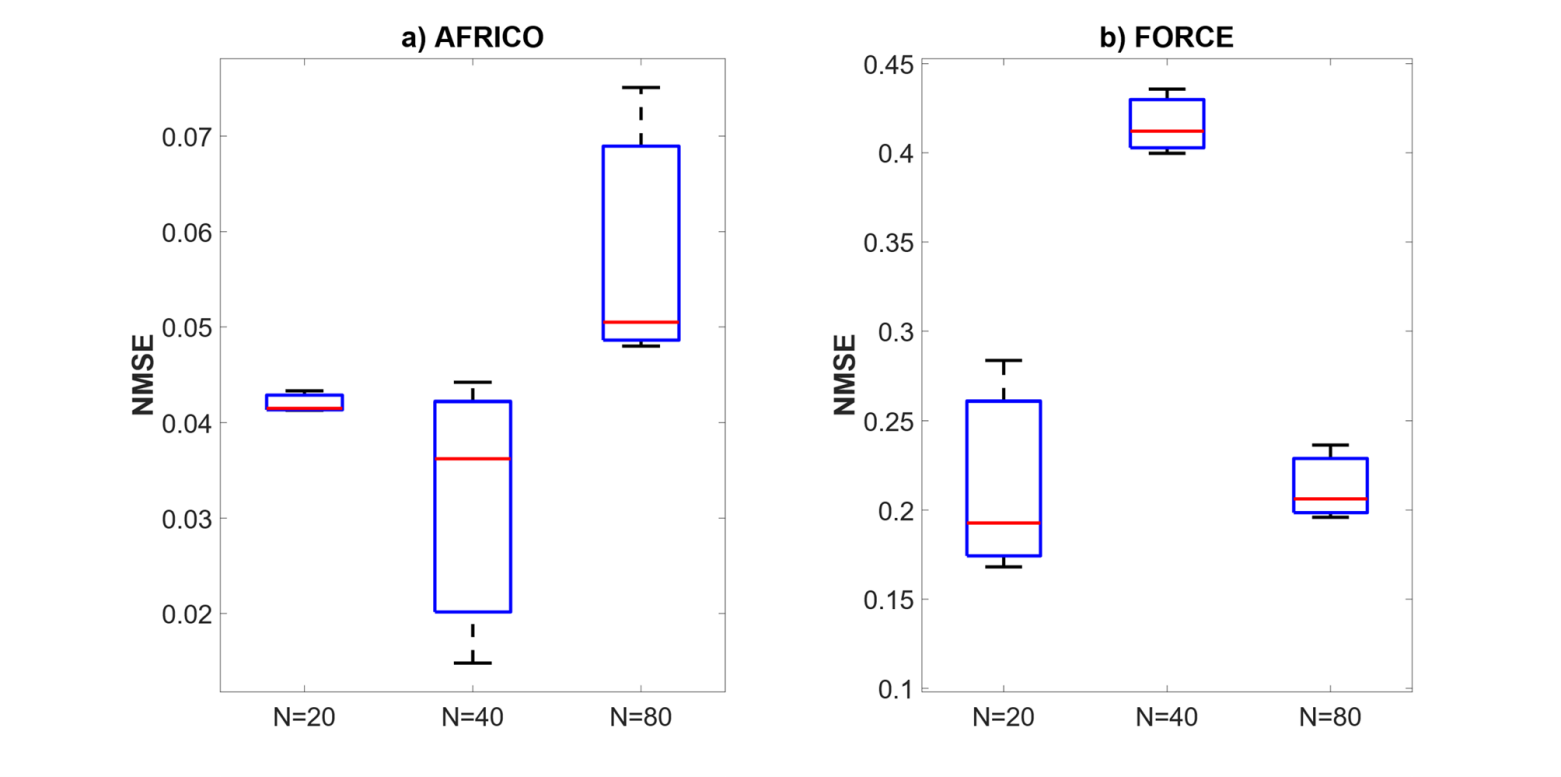
Example B: Boxplots showing the distribution of standard prediction errors for AFRICO and FORCE-trained ESNs across varying reservoir sizes, SNR of 50. Each box represents 100 independent simulations per configuration using **a** AFRICO-trained ESNs with state-feedback and **b** FORCE-trained ESNs with output-feedback.

Figure 9 compares AFRICO and FORCE performance on a nonlinear system, highlighting the advantages of adaptive state-feedback under increased model complexity. AFRICO achieves significantly lower NMSE across all tested reservoir sizes. In contrast, FORCE exhibits consistently higher errors, with only modest improvement at *N* = 80, and notably poor performance at smaller sizes. While AFRICO’s NMSE continues to improve with increasing reservoir size, its performance remains strong even at *N* = 20, reflecting robustness to model scaling and initialisation. These results confirm that AFRICO effectively captures nonlinear temporal dependencies through state-feedback modulation, outperforming FORCE in both accuracy and consistency. Similar trends have been observed for other SNRs (high noise − 10 and low noise − 50), as illustrated in Figs. 10 and 11.

Figures 5, 6, 7, 9, 10 and 11 show that increasing the number of reservoir neurons beyond a certain threshold does not yield further performance gains. From a control-theoretic standpoint, increasing reservoir dimensionality introduces additional dynamic modes. However, many of these modes may be weakly controllable or observable and therefore have a negligible effect on the input–output behaviour^37^. Such modes are typically disregarded by the sparse polynomial readout, so expanding the reservoir beyond a task-dependent threshold yields little benefit.

### Example C: Fruit fly photoreceptor data

The data used for this study are intracellular voltage recordings, measured in vivo from a fruit fly photoreceptor, in response to a naturalistic light stimulus as described in^33^. The light stimuli have been sampled at 2 kHz and low pass filtered. For this analysis, we selected the level 2 (L2) stimulus condition^33^. The state-feedback ESN architecture shown in Fig. 1b was trained on both linear and nonlinear reservoir configurations using the AFRICO algorithm. Training and validation datasets consisted of 3000 and 600 time points, respectively.

The reservoirs are fully connected and nonlinear, with hyperbolic tangent activation functions. Reservoir sizes range from 6 to 15 neurons across experiments. The recurrent weight matrices are initialised to have a spectral radius *ρ*(*W*) < 0.9 to ensure open-loop stability and compliance with the Echo State Property.

Input lags up to 7 were systematically varied as part of the optimisation, providing the reservoir with a delay-embedded representation of the stimulus. At each time step, the input was represented as a vector of current and lagged values, $${\mathbf{u}}(k)={\left[ {\begin{array}{*{20}{c}} {u_{ls}(k)}&{u_{ls}(k - 1)}&{...}&{u_{ls}(k - l)} \end{array}} \right]^T}$$, where ${u_{ls}}$ is the light-intensity stimulus and $l \in {\mathbb{N}}$ is the maximum lag. This embedding augments the reservoir with recent temporal context, analogous to time-delay embedding used in nonlinear system identification.

By extending the input space to include a finite history of past values, the ESN can capture more effectively systems with intrinsic delays or long-range dependencies, without increasing the size of the reservoir itself.

The polynomial readout was constructed from a candidate model set including all monomials up to degree three. Terms were selected using OFR with the ERR criterion, ensuring that only regressors contributing significantly to prediction accuracy were retained. This model order was found to provide a good balance between predictive accuracy and complexity for this task. For consistency with^33^, the output signal was mean-normalised prior to training.

**Fig. 12:**
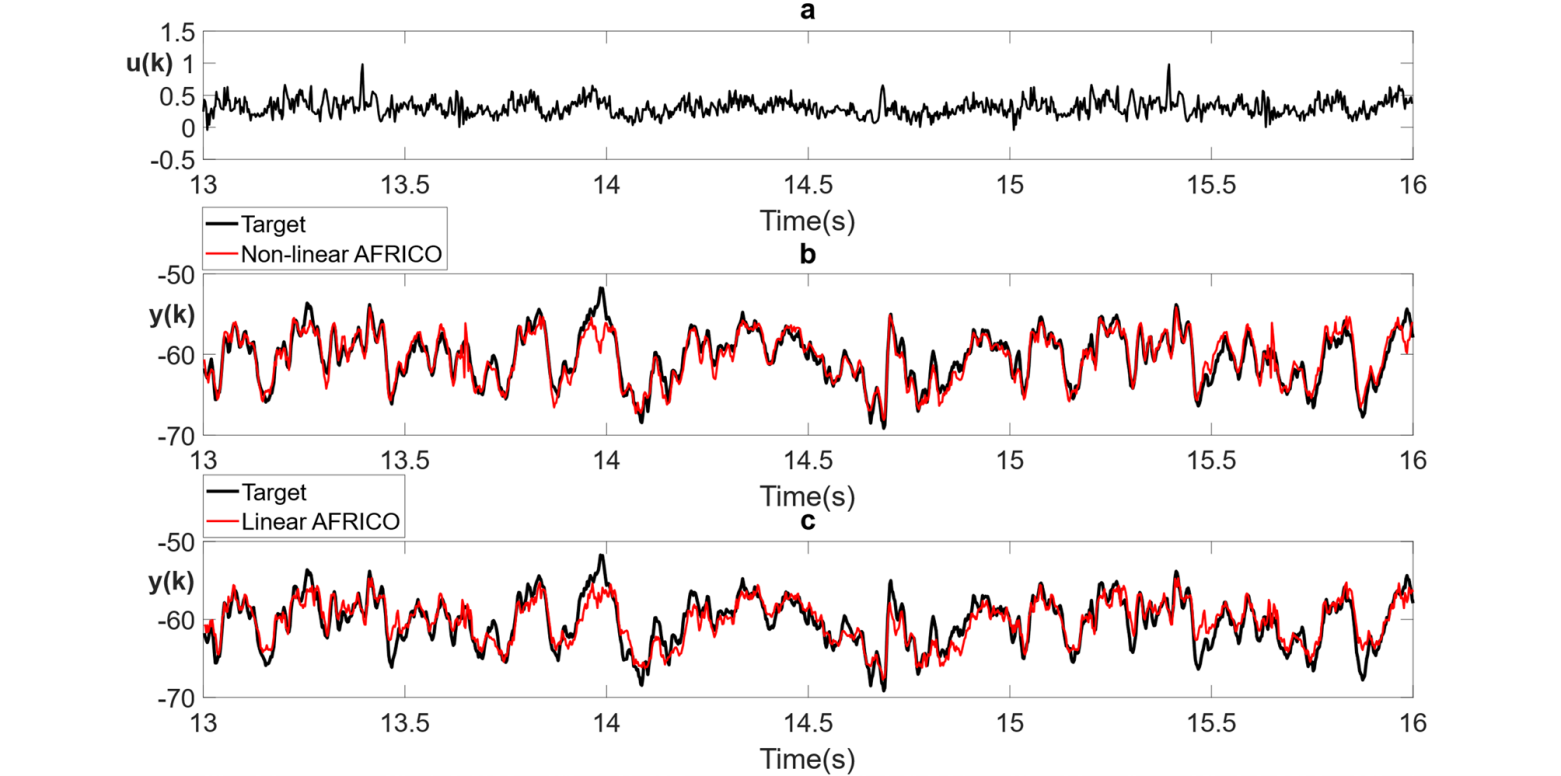
Example C: **a** Naturalistic light intensity input sequence, **b** model predicted outputs (red) for AFRICO-trained ESN with state-feedback and nonlinear reservoir (*N* = 11, maximum input lag *l* = 5) superimposed on the validation response (black) averaged over 10 simulations **c** model predicted output (red) for the AFRICO-trained ESN with state-feedback and linear reservoir (*N* = 13, maximum input lag *l* = 7) superimposed on the validation response (black) averaged over 10 independent simulations.

An illustration of polynomial terms selected across several runs for a nonlinear reservoir with *N* = 11 and input lag *l* = 5 is $\{ {x_1},x_{3}^{2},{x_2}{x_4},{x_1}{x_5},x_{6}^{2},{x_2}{x_3}{x_6},x_{1}^{3}\}$ corresponding to ~ 7–8% of the candidate model set of 364 terms, consisting of all monomials up to cubic order. The exact terms vary with random initialisation, but the sparse structure of the selected readout remains consistent. Here ${x_i}$ denotes the *i*th reservoir state.

Figure 12b and c illustrate the best-performing AFRICO-trained ESNs on the photoreceptor data set highlighting the impact of reservoir dynamics—linear versus nonlinear—on the performance of state-feedback ESNs applied to photoreceptor data. While both architectures use nonlinear readouts, the ESN with nonlinear reservoir (*N* = 11, *l* = 5) achieves a lower error (NMSE = 0.13), more accurately capturing the nonlinear processing that underpins photoreceptors’ capacity to compute phase congruency^33^ with a reported Relative Mean Squared Error of 0.83 (which translates to a NMSE of 0.7). By contrast, the best linear reservoir configuration (*N* = 13, *l* = 7) yields a mean NMSE of 0.17, indicating that internal nonlinearity enhances representational capacity, enabling more effective modelling of the temporal dependencies present in the photoreceptor data with fewer neurons and a lower-dimensional embedding of input dynamics.

Across 100 runs spanning input lags 1–7 and reservoir sizes 10–14, AFRICO produced consistently low and stable errors. Mean NMSE ranged from 0.14 to 0.26 (σ = 0.04–0.13), with minimum and maximum values from 0.03 to 0.53. A Wilcoxon signed-rank test yielded *p* = 0.03, confirming the performance advantage of the nonlinear setup.

For completeness, we also ran FORCE as a baseline on this task. FORCE consistently produced higher errors - average NMSE ≈ 0.3–0.4 across configurations (see Supplementary Fig. S3b, d).

This performance gap underscores the limitations of architectures with fixed linear dynamics and static nonlinear readouts, akin to Hammerstein models, which are known to be inadequate for systems with significant internal nonlinearities^38,39^. While both ESNs use nonlinear readouts, only the nonlinear reservoir can represent nonlinear state evolution, enabling richer internal dynamics and improved approximation of higher-order temporal dependencies.

### Example D: NARMA10 target system

The NARMA10 task is a widely used benchmark in reservoir computing^24^, designed to evaluate a model’s ability to capture nonlinear dynamics with long-range temporal dependencies, aspects directly addressed by AFRICO’s adaptive state-feedback formulation. The NARMA10 model is given by:

9 $$y(k+1)=0.3y(k)+0.05y(k)\sum\limits_{{i=0}}^{9} {y(k - i)} +1.5u(k)u(k - 9)+0.1$$

where *u* and *y* are the input and output respectively.

**Fig. 13:**
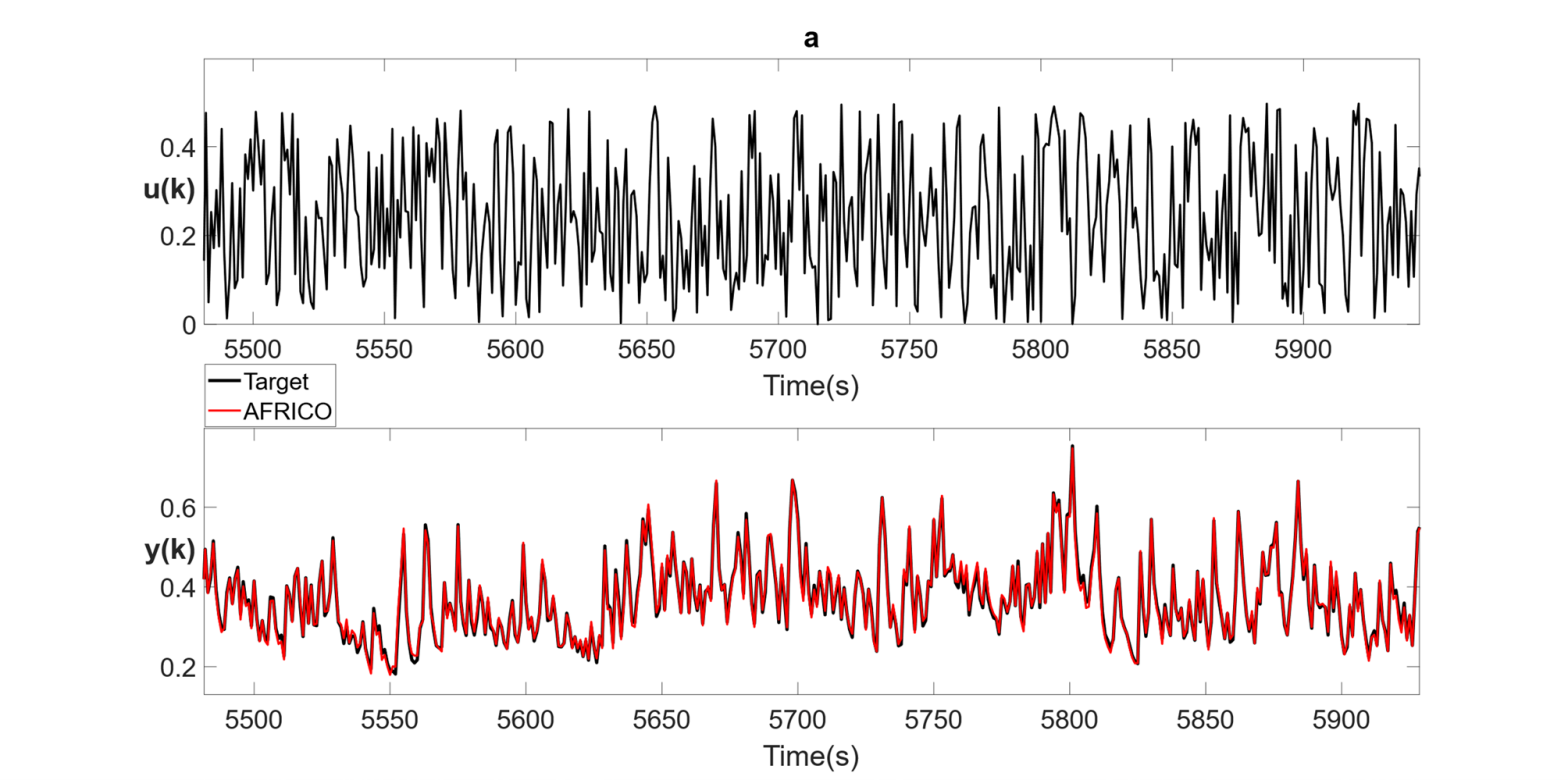
Example D: **a** input and **b** model predicted output for AFRICO-trained ESNs with state-feedback and nonlinear reservoirs (red), superimposed on the validation response (black), *N* = 52 neurons, SNR of 50., averaged over 100 simulations.

The input is drawn from a uniform distribution in the interval [0,1]. Network parameters follow standard initialisation across 100 independent instances, and fully-connected linear reservoirs with a uniformly distributed eigenspectrum and a spectral radius *ρ*(*W*) < 0.9, guaranteeing open-loop stability. Reservoir dimensions between 20 and 40 neurons were tested iteratively. The readout function was modelled as a polynomial function, with terms selected using the OFR algorithm from a candidate model set of polynomial regressors, up to cubic order. The training data consists of 3000 points, whilst the validation data consists of 1500 points (Fig. 13).

Across multiple runs with a nonlinear reservoir of *N* = 52, the OFR algorithm selected on average about 300 terms from 26,234 possible polynomial regressors, corresponding to roughly 1.1% of the candidate model set.

**Fig. 14:**
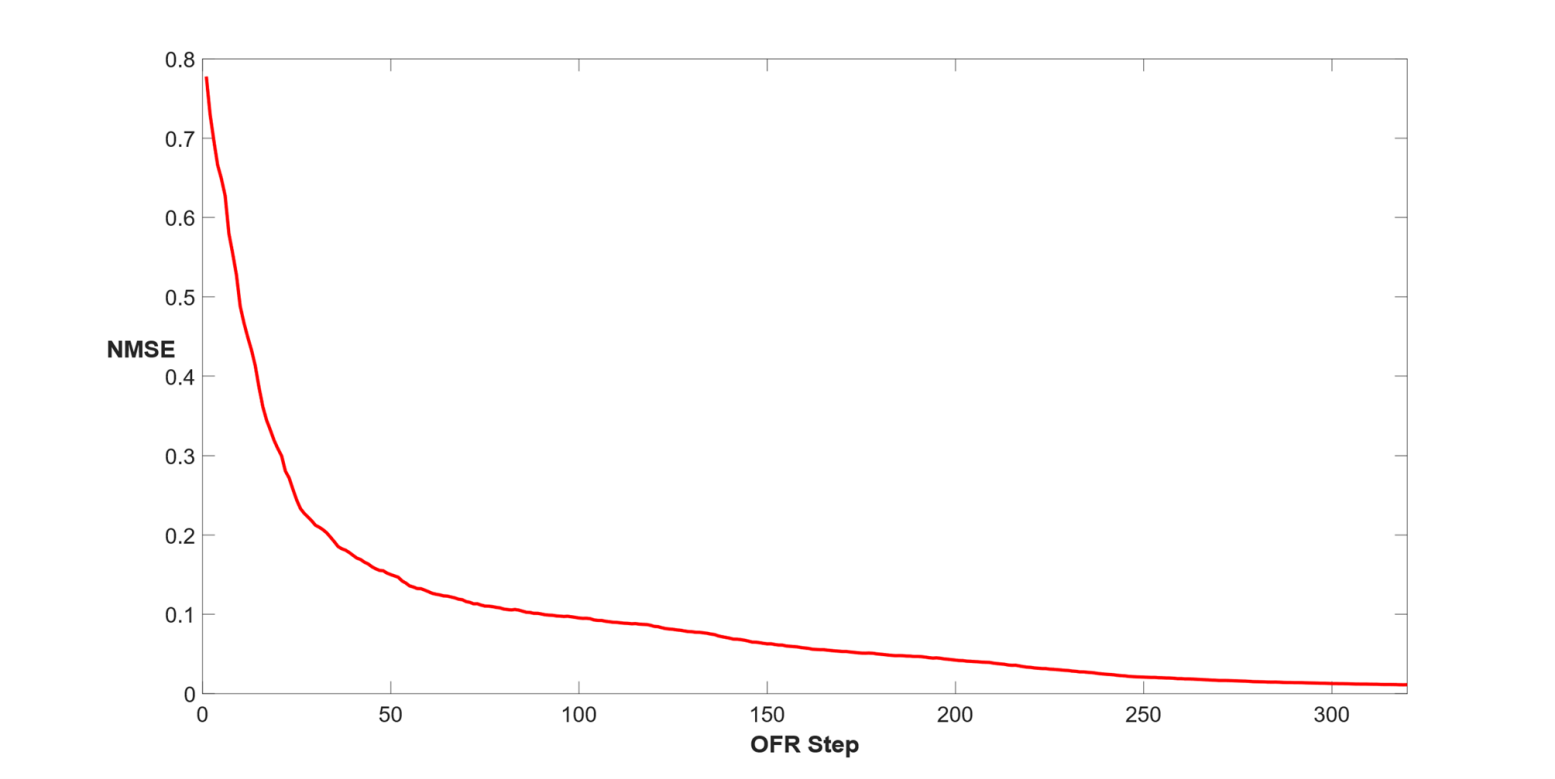
Example D: NMSE evolution during readout optimisation for NARMA10 benchmark using AFRICO (*N* = 52, SNR = 50).

The NMSE values for AFRICO-trained ESNs, for reservoir sizes ranging from *N* = 20 to 40 exhibit both low error and consistent performance. The mean NMSE ranges from 0.1271 for *N* = 20 to 0.022 for *N* = 40 indicating that all configurations yield comparably low prediction errors. The standard deviation is smallest for *N* = 40 (0.009), suggesting highly stable performance across delays and different noise configurations, while the highest variability is observed for *N* = 20 (0.134). The minimum and maximum NMSE values span from 0.01 to 0.25 across all settings. Extending the search to higher reservoir sizes we found that an ESN with *N* = 52 achieves a mean NMSE of 0.01 with a variance of 0.006. The evolution of the NMSE during training is illustrated in Fig. 14.

These results are significantly better than the results reported in^40^ using Distance-based Delay Networks (DDNs), which introduces inter-neuron delays based on spatial distance to improve ESN performance on the NARMA10. The reported best configuration, using 300 reservoir neurons, achieves an average NMSE of 0.0391, while relying on computationally intensive evolutionary tuning and a complex, delay-driven architecture.

**Fig. 15:**
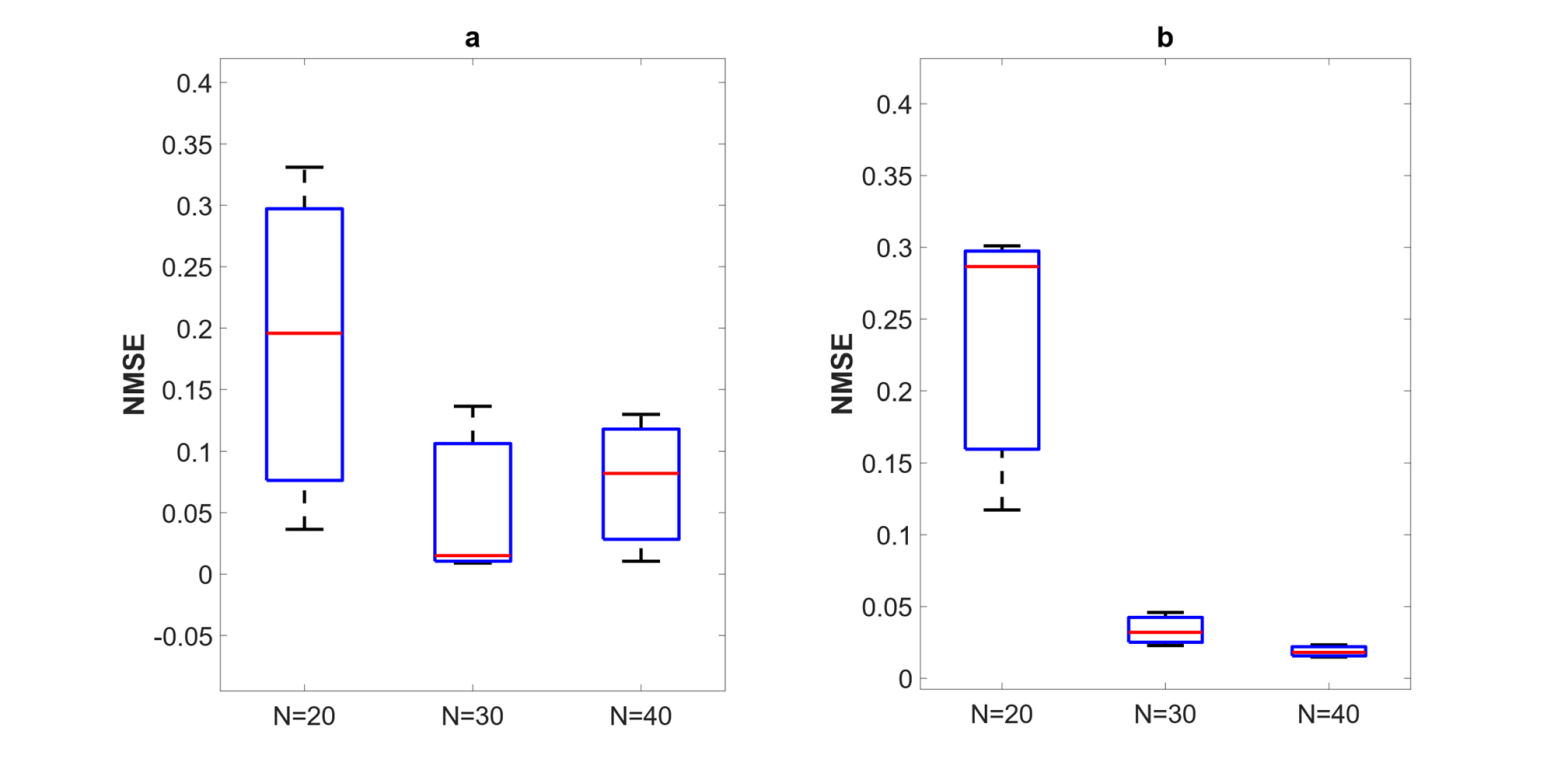
Example D: Boxplots showing the distribution of standard prediction errors for AFRICO ESNs across varying reservoir sizes. Each box represents 100 independent simulations per configuration using **a** linear reservoirs and **b** nonlinear reservoirs.

**Fig. 16:**
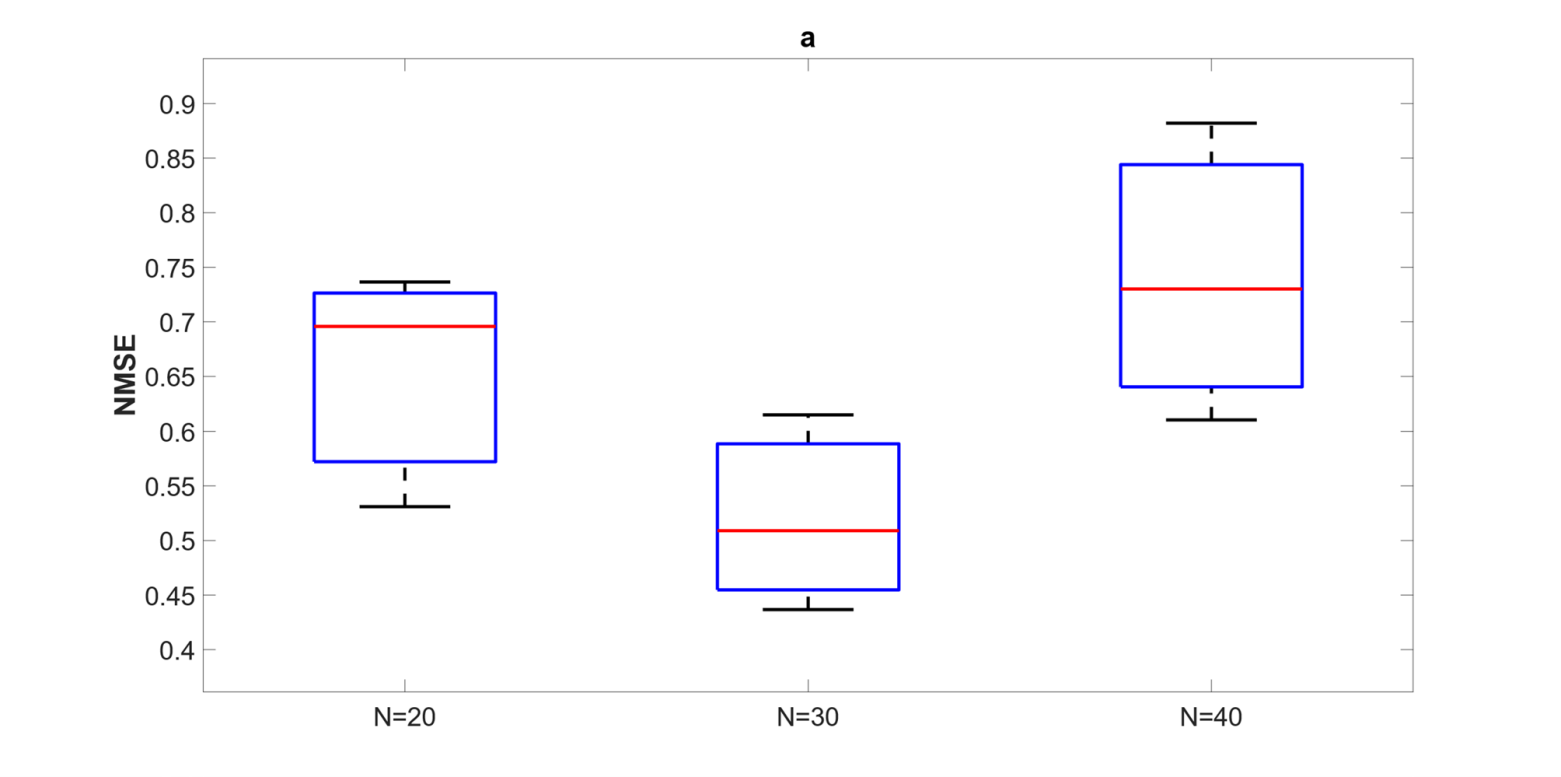
Example D: Boxplots showing the distribution of standard prediction errors for FORCE ESNs across varying reservoir sizes. Each box represents 100 independent simulations per configuration.

Figures 15 and 16 compare AFRICO and FORCE across varying reservoir sizes on the NARMA10 benchmark. With AFRICO, ESNs with linear reservoirs (Fig. 15a) achieve consistently lower errors than FORCE (Fig. 16) in both linear and nonlinear settings. When using nonlinear reservoirs, AFRICO delivers even stronger performance, with tightly clustered and significantly lower errors for reservoir sizes *N* = 30 and *N* = 40.

## Discussion

AFRICO is an adaptive training framework for Echo State Networks (ESNs) that optimises input weights and state-feedback gains using an Extended Kalman Filter (EKF) followed by the construction of a sparse polynomial readout via orthogonal forward regression (OFR). In contrast to traditional methods that rely on fixed output-feedback and restrict learning to the readout layer, AFRICO explicitly adapts input and feedback pathways to shape the reservoir’s internal dynamics.

This approach is motivated by the principle that a broad class of nonlinear systems can be locally approximated through appropriate state-feedback and output transformations^28^. The two-stage training process addresses two well-recognised limitations in standard ESN frameworks: the lack of mechanisms to adapt internal reservoir dynamics and the limited structure and interpretability of the output layer. The ESN architecture underpinning AFRICO satisfies the fading memory condition required by the universality results of Maass et al.^13^ and Maass & Sontag^28^, and thus it can approximate any causal time-invariant fading-memory functional. AFRICO defines a training scheme that enables such ESN architectures to realise their universal approximation capacity, provided the reservoir has sufficient dimensionality and the input and feedback pathways are appropriately adapted.

We provide a theoretical justification, grounded in classical system theory, that under dimensionality constraints, training both input and feedback pathways is necessary for an ESN to replicate the local input–output behaviour of a target dynamical system. While the dimensionality matching condition does not reflect typical ESN usage, it highlights that an ESN with adaptive input and feedback gains constitutes the minimal architecture capable of replicating the full system dynamics for a given state-space dimension. In practice many systems admit reduced-order models and AFRICO can operate effectively with lower-dimensional reservoirs to capture the dominant modes of the target dynamics.

Sussillo and Abbott^25^ also explored FORCE variants in which recurrent pathways were trained in addition to the readout. In one variant, a smaller feedback network was introduced, and FORCE learning was applied both to the readout weights and to the synapses projecting from the generator to this feedback subnetwork. In another variant, FORCE was applied directly to the generator’s recurrent synapses, which in a densely connected network amounts to updating essentially all recurrent weights, equivalent to full RNN training. However, the same global output error is broadcasted to update not only the readout but also the additional generator to feedback or the generator to generator synapses. While this heuristic update rule may enrich network dynamics, it does reflect the actual causal path from those synapses to the output. This does not provide a principled credit-assignment mechanism, so weight updates are not guaranteed to reduce the output error and may, in some cases, be detrimental. The results in^25^ and our own experiments show that this approach can lead to instability.

Empirical results across both synthetic and biological benchmarks show that AFRICO outperforms FORCE-trained ESNs not only in terms of accuracy, achieving up to 88% NMSE reduction, but also in robustness and compactness, often achieving optimal results with half the reservoir size. Notably, in the NARMA10 task, AFRICO outperforms distance-based delay ESN implementations^40^ that are known to perform well on this benchmark. These results support the theoretical insights in Lemma 1 and confirm that adapting input and feedback pathways overcomes structural limitations in ESNs with fixed reservoir dynamics.

While for a fixed reservoir size AFRICO trains more parameters than FORCE, its advantage comes from adapting input and state-feedback weights, which directly regulate reservoir controllability and dynamics.

AFRICO’s adaptive state-feedback mechanism modulates reservoir activity without altering internal weights, mirroring cortical circuit organisation and processing, where stable recurrent structures provide a substrate for computation, and feedback modulates neural activity in response to task demands. In the primary motor cortex (M1), for instance, context-dependent feedback shapes neural responses during movement^41^, while in the prefrontal cortex, feedback pathways seem to support cognitive flexibility and goal-directed behaviour^36^.

AFRICO achieves high predictive accuracy with reduced reservoir dimensionality, which is notable given findings that cognitive function often unfolds on low-dimensional neural manifolds^36^. This not only improves training speed and generalisation but also enhances robustness by suppressing noise and isolating principal modes of variation^42^.

The sparse readout structure further enhances both computational efficiency and interpretability. Empirically, fewer than 10% of reservoir-to-readout connections are typically retained to achieve high-accuracy. Importantly, this sparsity is a direct result of the OFR approach which selects only those regressors that provide a measurable reduction in prediction error, with cross-validation used as the stopping condition, resulting in a compact and task-adaptive connectivity pattern. This echoes findings from neuroscience that perceptual decisions and adaptive behaviours often depend on a select few neurons firing with temporally precise patterns^43,44^ and highlights AFRICO’s potential to probe functional specialisation within the reservoir^45^.

A limitation of AFRICO is the quadratic computational complexity of EKF with respect to the number of trainable parameters, which may hinder scalability for large reservoirs. Future work could explore reduced-rank EKF formulations to approximate parameter uncertainty in lower-dimensional spaces, structured or sparse feedback connections to reduce parameter count, and modular or hierarchical reservoir architectures that distribute computation across smaller interacting sub-networks.

### Methods

The exact numerical results presented here will vary slightly depending on the initialisation of the network, target system and on the precise software used to calculate them. The calculations were obtained using Matlab R2021b Update 7 (9.11.0.2358333) on an x86-64 CPU running Windows 10.

## Supplementary Information

Below is the link to the electronic supplementary material.

Supplementary Material 1

## Data Availability

The datasets generated and/or analysed during the current study are available in the Github repository, https:/github.com/CodrinLup/NatureESN.

## Code availability

All code is available in the Github repository, https://github.com/CodrinLup/NatureESN.
