# Supplementary material for: Adaptive state-feedback echo state networks for temporal sequence learning
Source: Sci Rep. 2026 Mar 16;16:13618. doi: 10.1038/s41598-026-42971-5 (PMC13121702; doi:10.1038/s41598-026-42971-5)
Supplement: Supplementary file 1 — Supplementary Material 1 [file 41598_2026_42971_MOESM1_ESM.docx]

**Supplementary note 1.**

Supplementary Note 1 provides the theoretical justification underlying Lemma 1 from the main text. Specifically, it contrasts the conditions under which ESNs with state-feedback and output-feedback can replicate the dynamics of a target system by matching their linearised transfer functions around an equilibrium point. The derivation highlights that matching both the dynamics and output of the target system generally requires training not only the readout, but also the input and feedback weights—underscoring the motivation for the AFRICO training framework. This analytical result supports the core claim that adaptive state-feedback is essential for improved system identification performance.

*Proof of Lemma 1.*

The target dynamical system is described by:

__ (1)

where __ the input, __ the target states and __ the output of the target, __ and __ the non-linear output function.

*ESN with state-feedback*

The goal is to train a non-linear *n*-dimensional discrete-time Echo State Network (ESN) with state-feedback to match the dynamics and output of the target system, where the ESN is described by the following state equations:

__ (2)

where __ is the reservoir state vector and __ is the ESN output,__ are the reservoir, input layer, state-feedback gain and readout connections, __, and the readout __ is a linear combination of *N* nonlinear basis functions __.

Without loss of generality, we assume that __, and that (**x***=0,**u*****=0) is an asymptotically stable equilibrium point for the ESN and target system.

Linearising the target system (1) around the equilibrium point yields:

__ (3)

where __ ,__and __ are the system, input and output matrices associated with the linearised system, respectively.

The transfer function representation of (3) is given by:

__ (4)

Linearising the ESN (2) around the equilibrium point yields:

__ (5)

taking into account that __and __.

The transfer function representation of (5) is given by:

__ (6)

Training the linear state-feedback ESN to exactly match the target system is equivalent to finding the weights __ that satisfy:

__ (7)

Equation (7) holds if and only if the linearised systems (3),(5) are *similar.* This implies the existence of a non-singular matrix *M* such that:

__ (8)

For simplicity, assuming __, the solution for the state-feedback weights is given by:

__ (9)

From (8) and (9) it follows that the ESN can match the dynamics and output of the target system only if the input, output and state-feedback weights are all trained.

*ESN with output-feedback*

Consider an ESN with output-feedback described by:

__ (10)

where__represents the fixed output-feedback weights.

Linearising the output-feedback ESN (11) around the equilibrium point yields:

__ (11)

Similar to the state-feedback case, for the output-feedback ESN to match the target in (4),

_ (12)_

where

__ (13)

is the transfer function representation of (11).

As before, (13) holds true if and only if there exists a non-singular transformation matrix *M* such that:

__ (14)

Assuming _,_ equation (14) leads to the following solution for the feedback weights:

__ (15)

This implies that the ESN with output-feedback weights can only match the target system if all the input, output and feedback weights are trained.

**Supplementary note 2 – Training methodology**

The objective of AFRICO is to train the input, output and state-feedback weights, as well as optimise the connectivity of the readout layer of a non-linear *n*-dimensional discrete-time Echo State Network (ESN) given in (2) to match the dynamics and output of the target system (1)

Given:

- Training data set: __.
- Validation data set: __

Stage 1 – Training the input and state-feedback weights

The Extended Kalman Filter (EKF) is applied to the training data set to estimate the state-feedback gain weights *W_fb_* and input layer weights *W_in_* of the ESN model (2).

To incorporate the trainable weights, define an augmented state vector that includes the reservoir state, the input and state-feedback weights:

__

where __and__are the vectorised input and state-feedback weights respectively.

Let

__

_and the diagonal Jacobian_

__

The augmented reservoir state transition equation is:

__

where *v*(*k*) is the augmented process noise, modelled as a zero-mean random vector with covariance *Q*.

The linearised augmented state transition equation is given by:

__

where

__

Quantities with a hat, denote the EKF estimates of the corresponding states and parameters at time *k*. These estimates are used to evaluate the Jacobians in the linearised model.

The augmented measurement (readout) equation is :

__

where __ is the measurement noise and the augmented output

The EKF prediction and correction equations are as follows:

Prediction step:

__

Correction step:

__

Updated estimate:

$$\begin{matrix} \hat{\bar{\mathbf{x}}}(k+1|k+1)=\hat{\bar{\mathbf{x}}}(k+1|k)+K(k+1)\varepsilon(k+1) \\ P(k+1|k+1)=\left(I-K(k+1)H(k+1)\right)P(k+1|k) \end{matrix}$$

Here *P*(*k*) denotes the state-error covariance matrix associated with the augmented state, *Q* is the process-noise covariance matrix, *K* the Kalman gain, *R* the measurement-noise covariance matrix and *S* the innovation (measurement-prediction) covariance matrix. The notation __ refers to the updated state estimate of the augmented state at time *k*, incorporating all measurements up to and including time *k*. The notation __ denotes the one-step prediction of the augmented state prior to observing the next measurement __. After incorporating _,_ the corrected estimate becomes __. The corresponding covariance matrices *P*(*k*+1|*k*) and *P*(*k*+1|*k*+1), and the predicted ESN output _,_ follow the same convention.

*Computational complexity of* __.

Stage 2 – Model selection and parameter estimation of a polynomial readout map *H*

In this stage, the readout layer is constructed using an Orthogonal Forward Regression (OFR) algorithm with an Error Reduction Ratio (ERR) criterion, as proposed in [26]. A candidate set of multivariate polynomial regressors is generated up to a specified order, and the most informative terms are greedily selected based on their contribution to reducing output prediction error. At each step, selected regressors are orthogonalised to ensure numerical stability and eliminate redundancy. The process continues until a cross-validation-based stopping criterion is met. This results in a compact, interpretable, and task-specific polynomial readout model. The method balances accuracy and complexity without relying on global optimisation or heavy regularisation.

**Step 1: Candidate polynomial model**

Define the polynomial readout function as

__

where

- __ is the vector of basis function weights
- *p_i_* are candidate polynomial basis functions of order *m*
- __are the readout layer weights
- *M_H_* is the total number of polynomial terms in the final model
- *N_H_* is the total number of candidate polynomial basis functions

Define:

- Polynomial order *m*
- Cross-validation error threshold __ (used to assess model adequacy)
- Error Reduction Ratio (ERR) threshold __

*Computational complexity of* __.

**Step 2:** **Generate state trajectories using the estimated state equations from Stage 1**

In this step, the Echo State Network state equations, incorporating the input weights __ and state-feedback weights __, estimated in Stage 1, are simulated to generate state trajectories corresponding to the input __.

*Computational complexity of* __.

**Step 3:** **Generate candidate polynomial model set**

Construct a candidate model set of all the regressors __ corresponding to a multivariate polynomial readout of order *m*. Each candidate polynomial basis function __is evaluated at every time step *k* over the training data to produce a vector __.

Initialise

- Iteration index __
- Best regressor index __
- Selected regressor set __

*Computational complexity of* __.

**Step 4**: **Perform Orthogonal Forward Regression (OFR) iteration**

Compute the Error Reduction Ratio (ERR) for each candidate regressor:

__

where __ represents the orthogonalised version of __ and __.is the training output vector.

Select the best regressor

__

Update the selected regressor set __ and increase the number of selected regressors __.

Compute the new vector of basis function weights

__

where __ is the design matrix which contains the selected orthogonalised regressors.

If __, proceed to Step 6; otherwise, continue to Step 5.

*Computational complexity of* __.

**Step 5: Orthogonalization Step**

After selecting __, the orthogonal basis is updated:

__

which ensures that newly selected regressors are linearly independent from previously selected ones.

Return to Step 4 to continue regressor selection.

*Computational complexity of* __.

**Step 6: Cross-Validation for model selection**

Compute validation error: __

If __ stop – final model found __, else

If __ continue to add regressors, return to Step 4.

Otherwise If __ consider increasing the polynomial order __ and return to Step 3.

*Computational complexity of* __.

**Supplementary Note 3. AFRICO vs FORCE performance on the photoreceptor dataset**

**a)**


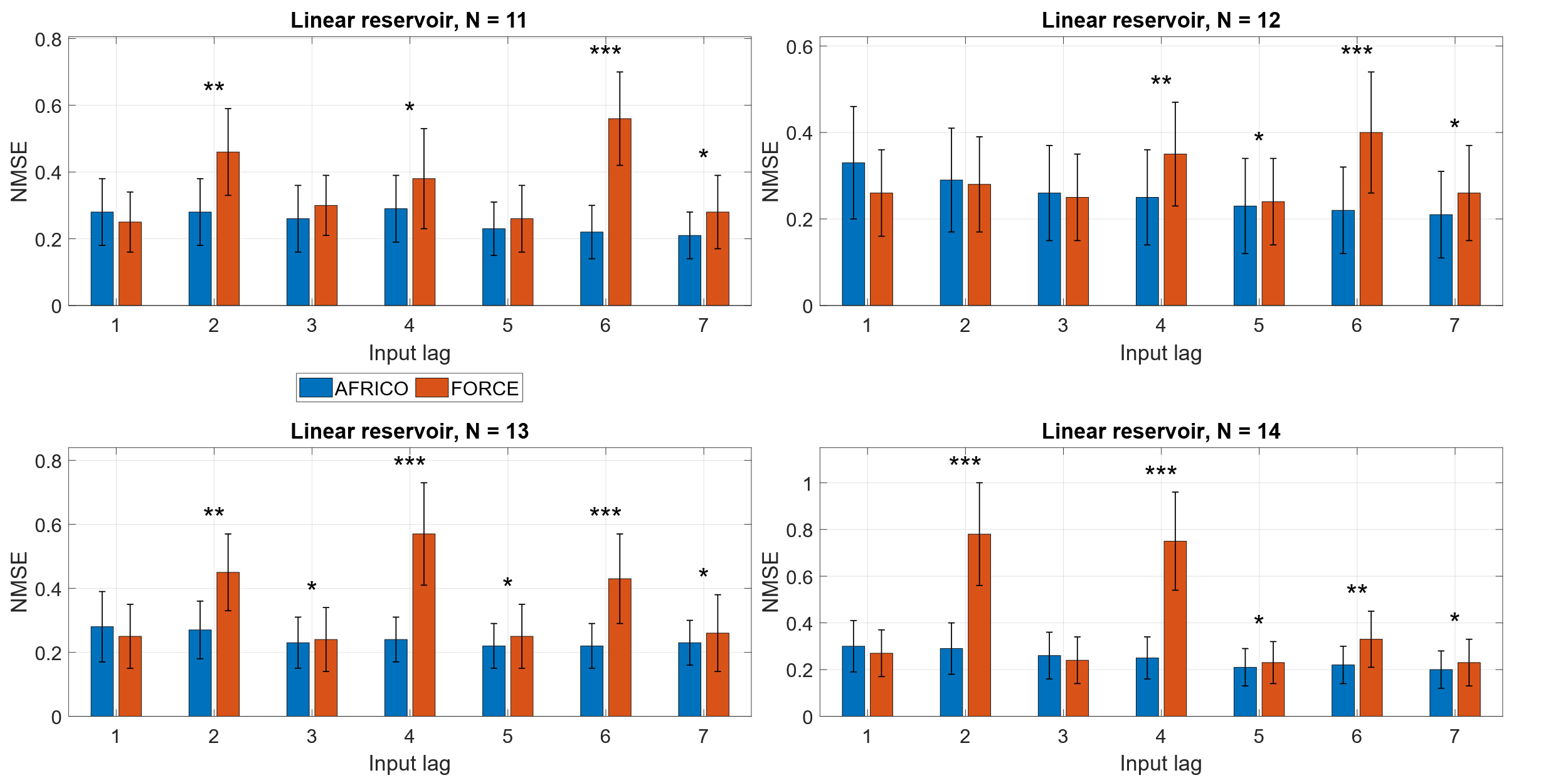


**b)**

**
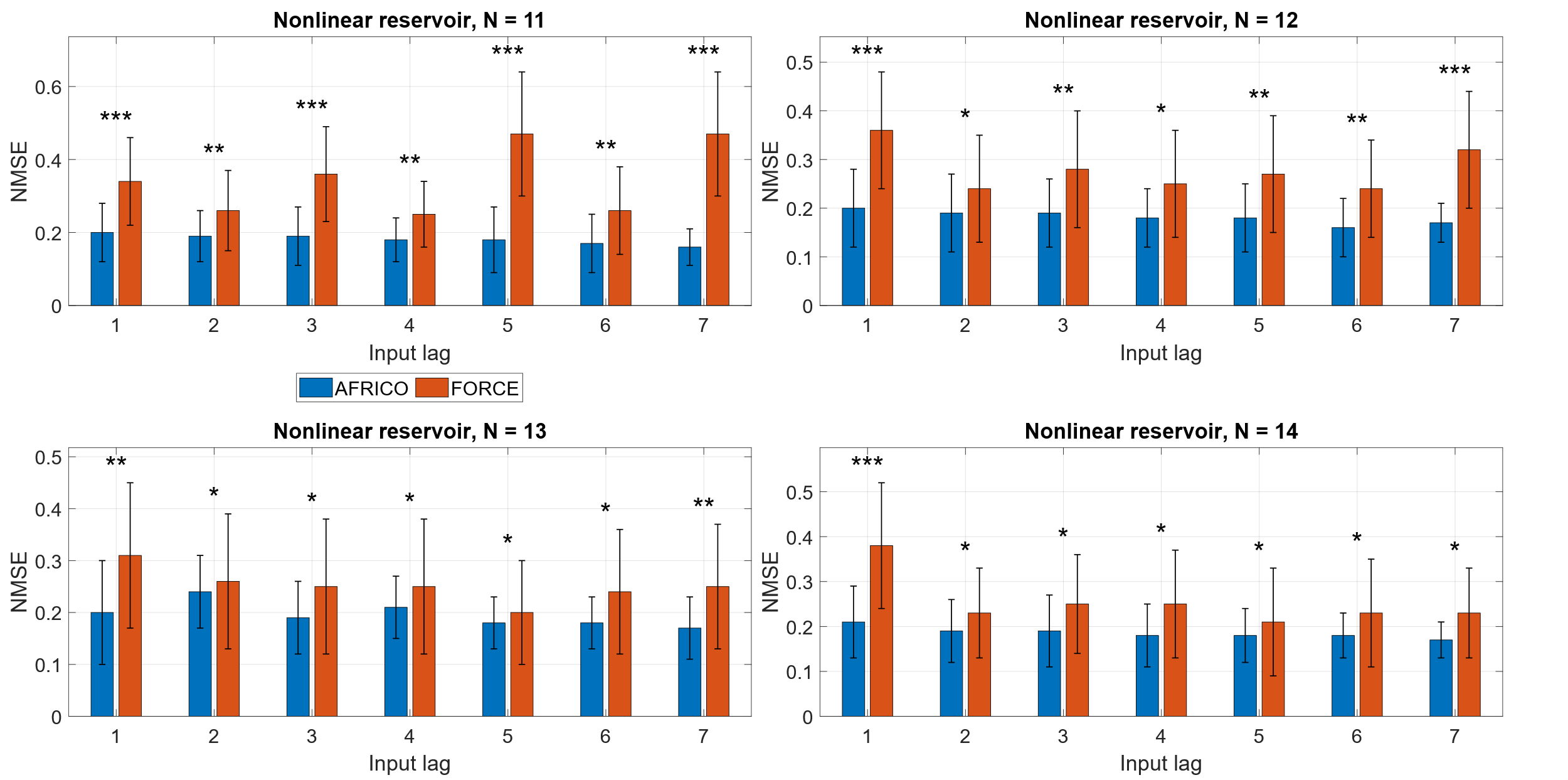
**

**Supplementary Figure S3. AFRICO vs FORCE performance on the photoreceptor dataset.** (a) AFRICO and FORCE with linear reservoirs, (b) AFRICO and FORCE with nonlinear reservoirs. Bar plots show the average NMSE across input lags for reservoir sizes *N* = 11–14, with each bar representing the mean over 100 simulations. Error bars denote the standard deviation across runs, and stars indicate levels of statistical significance based on paired tests between AFRICO and FORCE (*p < 0.05, **p < 0.01, ***p < 0.001). AFRICO achieves consistently lower NMSE with reduced variability across parameter settings, whereas FORCE exhibits higher errors and occasional severe deviations (average NMSE > 0.7), underscoring the robustness of AFRICO on real biological data.
